# Phosphorylation-Induced Self-Coacervation versus RNA-Assisted Complex Coacervation of Tau Proteins

**DOI:** 10.1021/jacs.4c14728

**Published:** 2025-03-12

**Authors:** Mohammadreza Allahyartorkaman, Ting-Hsuan Chan, Eric H.-L. Chen, See-Ting Ng, Yi-An Chen, Jung-Kun Wen, Meng-Ru Ho, Hsin-Yung Yen, Yung-Shu Kuan, Min-Hao Kuo, Rita P.-Y. Chen

**Affiliations:** † Taiwan International Graduate Program in Interdisciplinary Neuroscience, National Taiwan University and Academia Sinica, Taipei 115, Taiwan; ‡ Institute of Biological Chemistry, Academia Sinica, No. 128, Sec. 2, Academia Road, Nankang, Taipei 115, Taiwan; § Institute of Biochemical Sciences, National Taiwan University, No. 1, Sec. 4, Roosevelt Road, Taipei 106, Taiwan; ∥ Department of Biochemistry and Molecular Biology, Michigan State University, 603 Wilson Road, Room 401, East Lansing, Michigan 48824, United States; ⊥ Neuroscience Program of Academia Sinica, Academia Sinica, No. 128, Sec. 2, Academia Road, Nankang, Taipei 115, Taiwan

## Abstract

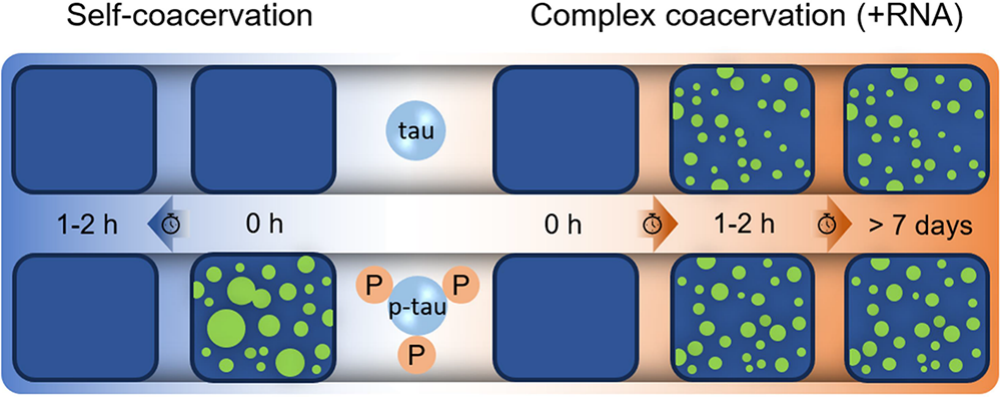

In this study, the role of phosphorylation in the liquid–liquid phase separation (LLPS) of tau, the underlying driving forces, and the potential implications of this separation on protein conformation and subsequent protein aggregation were investigated. We compared in vivo-produced phosphorylated tau (p-tau) and nonphosphorylated tau under different coacervation conditions without adding crowding agents. Our findings revealed that spontaneous phase separation occurs exclusively in p-tau, triggered by a temperature shift from 4 °C to room temperature, and is driven by electrostatic and hydrophobic interactions. The p-tau self-acervation is reversible with temperature changes. Native mass spectrometry detects only two to nine phosphate groups per p-tau molecule, highlighting the impact of phosphorylation on tau’s structural flexibility. Cross-linking mass spectrometry showed fewer long-range contacts in p-tau, suggesting a looser conformation induced by phosphorylation. Phosphorylation-induced LLPS and RNA-induced LLPS occurred at different timeframes. However, neither tau nor p-tau formed fibrils without the addition of dextran sulfate or RNA as inducers. Using human kidney epithelial cells expressing the tau R domain fused with fluorescent proteins as reporter cells, we observed aggregates in the nuclear envelope (NE) only in the cells treated with LLPS-state p-tau, which correlates with NE occurrences reported in Alzheimer’s disease brain sections. These findings provide deeper insights into the impact of phosphorylation on tau aggregation through an intermediate condensation phase, offering novel perspectives on neurodegenerative disease mechanisms.

## Introduction

Many proteins can coalesce into membrane-less assemblies with liquid-like characteristics, a process known as liquid–liquid phase separation (LLPS).^1^ LLPS results in the formation of granules or droplets with densely packed macromolecules, driven by transient interactions among molecules with multivalent, intrinsically disordered regions.^2,3^ LLPS can be classified as self-coacervation (SC) or complex coacervation (CC). SC is triggered by electrostatic interactions between differently charged domains of the protein, while CC is driven by the absorption or repulsion of similar or oppositely charged polymers (polyanions), such as nucleic acids.^4−6^ Unstructured proteins are flexible enough, making them susceptible to phase separation.^7^

Tau is a microtubule-associated protein. Tau binding helps to stabilize microtubules, but its pathological self-association is the cause of many neurodegenerative diseases. Multiple strands of evidence indicate that the oligomerization and aggregation of tau are cytotoxic to neurons.^8−12^ However, the mechanisms driving tau’s transition from a normal monomer to pathological forms remain unclear. The pathology of tau is believed to commence with its separation from microtubules, possibly triggered by hyperphosphorylation or a reduced dephosphorylation rate.^13,14^ Phosphorylation is the most common modification in tau, accounting for up to approximately 20% of its post-translational modifications. Introducing negative charge(s) can alter tau’s function, degradation, and aggregation.^15,16^ In the brain of a normal individual, tau, featuring a phosphate-to-protein ratio of approximately 2–3, promotes the assembly of tubulin and maintains the stability of microtubule structures. Conversely, the hyperphosphorylated form of tau (p-tau), characterized by a phosphate-to-protein ratio of 6–8, is present within the distinctive neurofibrillary tangles observed in the brains of patients suffering from Alzheimer’s disease (AD).^17−20^ Tau malfunction is largely attributed to extensive hyperphosphorylation occurring at different residues, leading to instability and disassembly of microtubules,^21,22^ and, ultimately, dystrophic neurites.^23^ Hyperphosphorylation transforms tau into a form that readily aggregates, resulting in a change in its aggregation propensity and cytotoxicity, which are central to the development of diffusible pathology in conditions such as AD and other tauopathies, ultimately contributing to cellular demise.^22,24^

Tau’s intrinsically disordered nature and its uneven charge distribution throughout its structure enable it to undergo LLPS when exposed to crowding agents, such as polyethylene glycol (PEG), dextran, or Ficoll.^25−28^ Despite the common existence of LLPS with intrinsically disordered proteins (IDPs), the physical principles that dictate its formation or characteristics are not uniform. The intermolecular electrostatic charge–charge and dipole–dipole interactions between the positively charged middle region and negatively charged N-terminal regions of tau are considered the primary causes of LLPS.^29^ However, studies on PEG-induced LLPS have highlighted the role of hydrophobic interactions, demonstrated by PEG’s capability to remove water from the condensed phase core.^30,31^ Among all molecules inducing LLPS, the pathological interaction of RNA and tau is implicated in tauopathies.^32^ Tau aggregates purified from HEK293 tau biosensor cells and P301L mouse brains are enriched for snRNAs and snoRNAs.^33^ Tau colocalizes with RNA-binding proteins in stress granules.^34^ Nonphosphorylated tau appears in puncta localizing to the nucleolar, and glutamate-induced stress results in nucleolar tau redistribution and an increase of nuclear p-tau.^33,35^ RNA also plays an adverse role in microtubule polymerization and conformational changes.^36^ Moreover, tau aggregates with the help of RNA.^37^ Based on opposite charge attraction, RNA associates with tau, creating a heterotypic CC.^6,28,38^ Contrary to electrostatically driven LLPS, high salt concentration can also promote phase separation, leading to tau dehydration and fibrillization through hydrophobic interactions.^30^

The major features of the tau pathological conformation remain unclear. It has been proposed that tau can adopt two distinctive structures: a paper-clip-like conformer, formed because of the proximity of two ends to the middle part, and an open conformer with an exposed middle part with two terminals projected away from the middle, either when tau is bound to microtubules or when it is present in paired helical filaments.^39,40^ Therefore, the paper-clip-like conformation, by shielding the central domain, may protect the protein from fibrillization.^41^ Truncated tau without terminals with higher repeat domain (RD) accessibility resulted in better fibrillization than full-length tau.^42,43^ In a heparin-induction experiment, researchers found the structural difference between the inert tau monomer (M_i_) and the heparin-induced, seed-competent tau monomer (M_s_), and reported that M_s_ can act as a template to generate distinct tau “strains.”^44,45^ This diversity intrigued us to closely examine tau conformation when the protein undergoes LLPS in the normal or phosphorylated state. According to the literature, tau–RNA complexes can alter the tau conformation, recognized as a pretangle tau epitope in mouse models of tauopathy.^36^ Still, it is unclear whether fibrillization results from LLPS as an intermediate facilitation step or whether they are independent processes. In this study, we aimed to investigate tau coacervation and its relationship with tau phosphorylation and RNA binding. Tau has six isoforms (0N3R, 0N4R, 1N3R, 1N4R, 2N3R, and 2N4R) resulting from mRNA splicing.^46^ The 1N4R form was used in this study because it is the most abundant isoform in adults.^47^

## Results and Discussion

### Expression of P-tau in *E. coli* Using the Protein Interaction Module-Assisted Function X System

**Figure 1:**
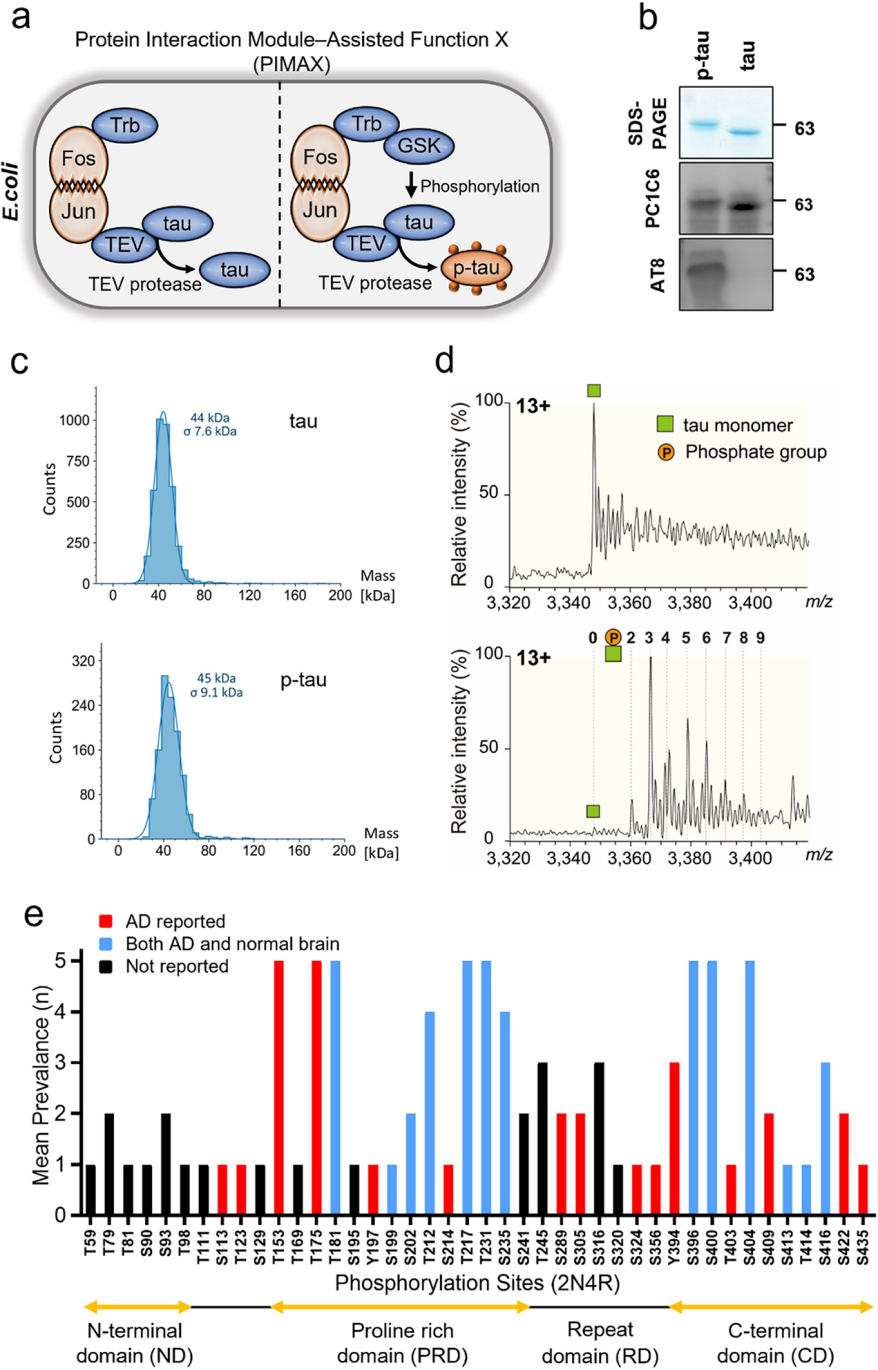
Tau/p-tau purification and characterization. (a) Schematic presentation of the PIMAX system used for the production of tau and p-tau. Trb: thrombin. (b) SDS-PAGE analysis of purified tau and p-tau, visualized by Coomassie Brilliant Blue staining and Western blotting. The Western blot used tau-1 (PC1C6 clone) for detecting total tau and AT8 for detecting p-tau. (c) Molecular mass distributions of tau and p-tau using mass photometry (TwoMP). (d) Zoom-in spectra for tau and p-tau ions at charge 13+ from native mass spectrometry data, highlighting the quantification of post-translational modifications in p-tau relative to tau. (e) Mapping phosphorylation of p-tau using LC-MS/MS. The *Y*-axis represents the prevalence observed in five independent replicates.

The protein interaction module-assisted function X (PIMAX) system uses leucine zipper protein–protein interaction modules (PIMs), derived from Fos and Jun proteins, to establish an association between a protein of interest (tau) and its “facilitator” (glycogen synthase kinase 3β [GSK3β]). Through the action of PIMs, tau and GSK3β are brought into proximity, thereby facilitating tau phosphorylation inside the bacterial cell.^24,48^Figure 1a shows a schematic of the PIMAX system with (right) and without GSK3β (left). Tau and p-tau were purified by using a strong cation-exchange chromatography column to eliminate tau-bound nucleic acids. Subsequently, the proteins were subjected to a size exclusion column to separate proteins of different sizes. The separated proteins were visualized on 15% sodium dodecyl sulfate-polyacrylamide gel electrophoresis (SDS-PAGE) (Figure S1a). The UV spectra of both tau and p-tau display peaks at 275 nm but not at 280 nm, consistent with tau containing five tyrosine residues and no tryptophan (Figure S1b). Further validation was conducted using the Tau-1 (PC1C6 clone) antibody against total tau, which exhibited reactivity with both tau and p-tau. Conversely, only p-tau exhibited immunoreactivity to the AT8 monoclonal antibody, specifically targeting the Ser202 and Thr205 sites. The SDS-PAGE analysis, as shown in Figure 1b, revealed that p-tau exhibited slower mobility than tau due to phosphorylation.

### Protein Identification and Phosphorylation Site Distribution in Different Domains of the Protein

Tau and p-tau were analyzed using mass photometry (TwoMP, Refeyn, UK) to measure their molecular masses and determine whether they were monomeric.^49^ The measurements for both proteins were conducted, yielding values of 44 ± 7.6 kDa for tau and 45 ± 9.1 kDa for p-tau (Figure 1c). Unlike conventional mass spectrometry methods, which use denaturing conditions, native mass spectrometry preserves the natural structure, conformation, and even noncovalent interactions of biomolecules.^17^ The use of native mass spectrometry facilitated the precise quantification of post-translational modifications on the protein, enabling an accurate mass measurement without altering its natural state. The native mass spectrum of tau (Figure S2 and upper panel in Figure 1d) illustrated a single peak of tau protein with the monomer molecular mass calculated as 43,511.98 ± 0.36 Da, while the spectrum of p-tau (lower panel in Figure 1d) indicated that p-tau, produced in *E. coli* using the PIMAX system, carried between 2 and 9 phosphate groups.

Several studies have highlighted the specific tau phosphorylation sites in the cerebrospinal fluid of patients with AD.^50,51^ Phosphorylation sites such as T181, S202, T205, T217, T231, and S235 (numbers based on the 2N4R isoform of tau) are currently used as biomarkers for AD diagnosis.^52−55^ In our study, the phosphorylation sites on p-tau were identified using liquid chromatography with tandem mass spectrometry (LC-MS/MS). This method allowed for the characterization of the phosphorylation pattern, which was then compared to previously reported patterns in AD and normal brains.^22,56^Figure 1e illustrates the phosphorylation pattern and its frequency. Not all phosphorylation sites identified through tandem mass spectrometry were located within a single protein molecule, as the native mass data in Figure 1d indicate only 2–9 phosphate groups per protein molecule. The red color represents GSK3β phospho-sites already reported in AD brains, blue shows sites reported in both AD and control brains, and black represents sites never reported (tau phosphorylation sites are listed on Prof. Diane Hanger’s Web site: https://bit.ly/2JyZTbS). Our data indicated that tau phosphorylation in *E. coli* by GSK3β occurred mainly at the sites reported in AD brains. Five AD biomarkers (phosphorylation at T181, S202, T217, T231, and S235) can be found in p-tau. Tau protein comprises four domains: N-terminal domain (ND), proline-rich domain (PRD), repeat domain (RD), and C-terminal domain (CD). Intriguingly, most phosphorylated sites were identified within the PRD and CD. Our findings revealed a dynamic distribution of phosphorylation across the tau molecule by the kinase GSK3β, particularly in PRD and CD. It should be noted that GSK3β phosphorylation is influenced by prior phosphorylation on its substrate.^57^ As our PIMAX system lacks other kinases, the phosphorylation site identified in Figure 1e may not fully represent the GSK3β-driven tau phosphorylation observed in cellular contexts.

### Spontaneous LLPS of P-tau Was Temperature- and Protein Concentration-Dependent

**Figure 2:**
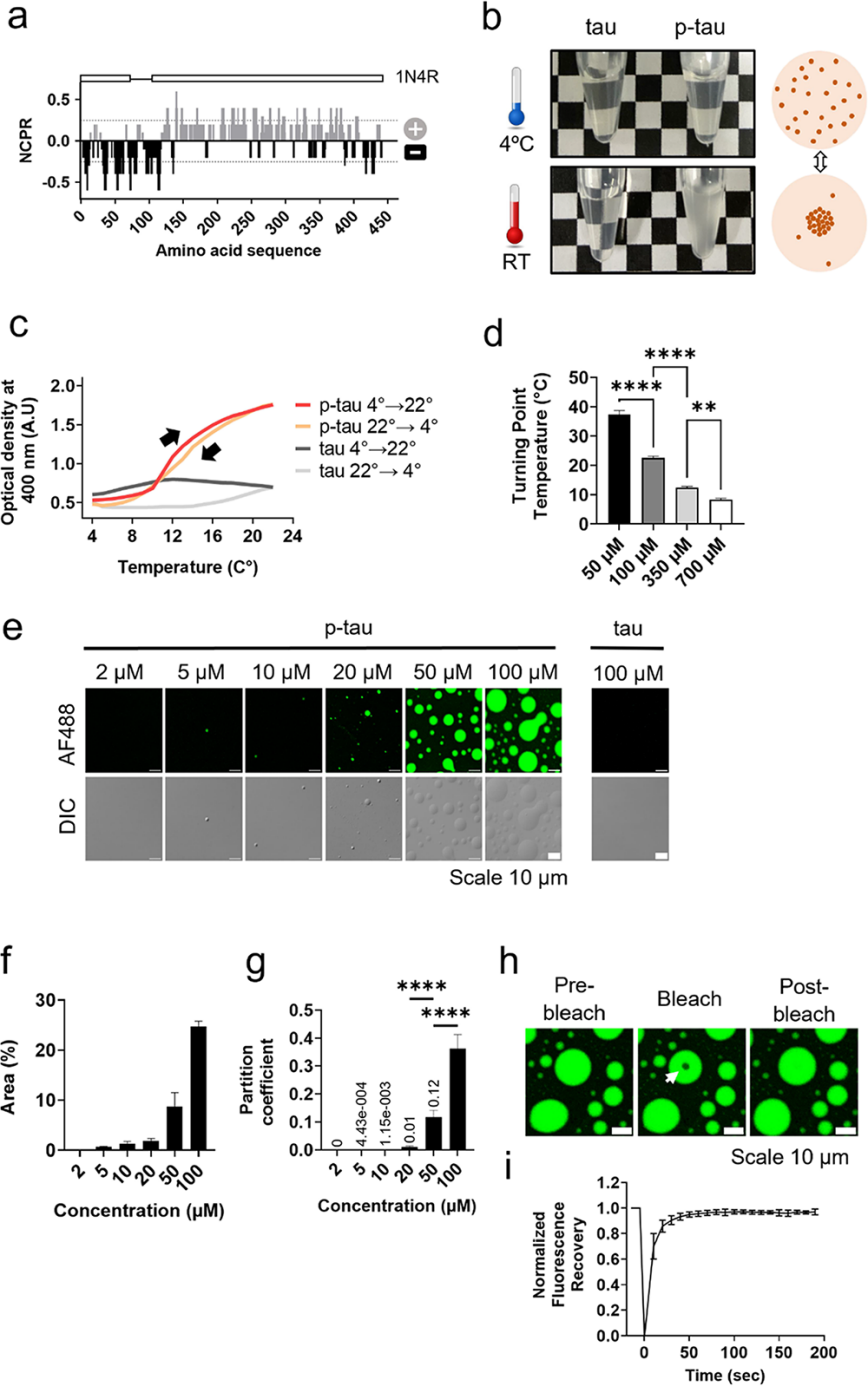
Temperature-and concentration-dependent alterations in tau LLPS through phosphorylation. (a) Asymmetrical charge distribution in tau. The black and gray colors represent negatively and positively charged amino acids, respectively. NCPR, net charge per residue (by EMBOSS) (b) Transitioning from 4 °C to room temperature (RT), tau and p-tau at a concentration of 350 μM in a solution of 20 mM Tris and 20 mM NaCl at pH 7.4 displayed differences in visible turbidity. (c) Temperature-dependent turbidity changes in p-tau were assessed by measuring light scattering at 400 nm with a heating rate of 1 °C/min. The curves are the average of three technical repeats. (d) Turning point temperature (°C) was measured at various p-tau concentrations. Data are shown as mean ± SD (*n* = 3) and analyzed by one-way ANOVA with Tukey multiple comparisons test. *P < 0.05, ****P < 0.0001. (e) Concentration-dependent droplet formation of p-tau was observed using confocal microscopy. At concentrations of 5 μM or above, p-tau exhibited LLPS, whereas high concentrations of tau did not show phase separation. Phase separation was visualized by mixing a 1:100 ratio of AF488-labeled protein with unlabeled protein. (f) Quantification of p-tau condensation area percentage (%) was measured at various p-tau concentrations. (g) Quantification of the LLPS partition coefficient (*K*) at various p-tau concentrations. Data in (f) and (g) are shown as mean ± SD (*n* = 3) and analyzed by one-way ANOVA with Tukey multiple comparisons test. ****P < 0.0001. (h) The moving dynamics of p-tau inside the droplets were determined using the FRAP test. The fluorescent images before bleaching, immediately after bleaching, and recovered after bleaching are shown. (i) Plot of time-dependent fluorescence recovery of the bleached area in (h).

Tau exhibits an uneven charge distribution with positively charged residues mainly clustering in the middle of the sequence and negatively charged residues clustering at the two terminal regions (Figure 2a). This biophysical trait, attributed to the intrinsically disordered sequence and uneven charge distribution of tau, crucially affects its susceptibility to LLPS.^29,30,38,58^

Given the close association between hyperphosphorylation of tau and neurodegeneration in AD and other tauopathies, we hypothesized that hyperphosphorylation contributed to tau aggregation through the promotion of LLPS. Indeed, p-tau formed condensed droplets that scattered light immediately after incubating the p-tau solution at room temperature, resulting in turbidity visible to the naked eye (Figure 2b). This phase separation phenomenon was reversible; the p-tau solution became clear again when cooled to 4 °C (Figures 2c and S3a). Most importantly, no crowding agent is involved in this process. This contrasts with other studies that found tau LLPS required a crowding agent, such as PEG or Ficoll.^25^ Different from p-tau, tau remained clear under both temperature conditions. This experiment demonstrated that phosphorylation can alter tau’s condensation behavior. A higher temperature was required when the p-tau concentration was lower, indicating that protein association was driven by hydrophobic interactions (Figure 2d). Throughout the examined concentration ranges, p-tau consistently exhibited higher turbidity compared with tau (Figure S3b). Interestingly, the relationship between temperature and tau pathology is complex. One study reported that hibernation enhanced paired helical filaments (PHFs)-like phosphorylation of tau, which is reversed upon arousal, suggesting an adaptive mechanism associated with neuronal plasticity in hibernating animals.^59^ Another human study pointed out that lower body temperatures in older adults may correlate with increased tau pathology.^60^ Conversely, elevated ambient temperature in a mouse model increased tau phosphorylation and exacerbated cognitive function and AD pathologies.^61^ In our study, the transition temperature for 50 μM p-tau between monomeric and LLPS states was near body temperature (Figure 2d), implying that a lower body temperature may inhibit p-tau coacervation, potentially contributing to neuroprotection. These findings highlight the intricate role of temperature in tau pathology, which may vary depending on the pathway and stage of disease progression, warranting further investigation.

To visualize the LLPS droplets under a confocal microscope, the proteins were labeled with an Alexa Fluor 488 (AF-488). The efficiency of the labeling was then examined using gel electrophoresis (Figure S3c). The degrees of labeling for tau and p-tau were found to be close, with values of 1.95 ± 0.03 and 1.9 ± 0.05, respectively. This indicates that nearly two dye molecules were attached to each protein molecule. To minimize any negative impacts of labeling on the experiment, the droplets were visualized by using a molar ratio of 1:100 between AF488-labeled and unlabeled proteins. Phase separation was exclusively observed in p-tau, not in tau, under room temperature conditions when examined using a confocal microscope (Figure 2e). As the protein concentration increased, both the droplet size (Figure 2e) and the coverage area (Figure 2f) increased. This increase in the coverage area demonstrated concentration-dependent LLPS for p-tau adsorption in vitro. Consistent with this, the ratio of the dense-to-dilute phase, known as the partition coefficient (K), also increased with higher protein concentrations (Figure 2g).

The Fluorescence recovery after the photobleaching (FRAP) assay was used to confirm the dynamic movement and liquidity of the protein within the droplet. p-tau exhibited almost complete recovery after being bleached in the circular region of interest (ROI) with a 2-μm diameter (Figure 2h–i). The diffusion coefficient, calculated as 0.144 μm^2^/s ± 0.022, was derived from the equation proposed by Axelrod and Soumpasis.^62^

Meanwhile, condensate coalescence refers to the merging or coming together of individual droplets in an LLPS system. Recording the droplet coalescence could provide insights into the movement dynamics of p-tau molecules inside the droplets. Essentially, permanent aggregation is rigid and unable to move or merge, but in this study, a two-regime LLPS could move dynamically (Movie 1). A concentration of 100 μM p-tau was studied and framed every 10 s for 5 min. A genuine LLPS phenomenon is known for its propensity to precipitate in vitro. In this study, the key characteristic of surface wetting was observed for p-tau, similar to the results recorded for tau in a crowding environment.^63^ In practice, gravity caused the droplets to descend immediately after the onset of the phase separation process. This phenomenon was recorded every 5 min by capturing images at a depth of 70 μm within the solution at every 5 μm of the Z-stack (Movie 2). Droplet precipitation was quantified using the mean area (μm^2^) across the Z-stack (Figure S3d).

### P-tau Forms a Different Conformation from Tau under the LLPS Conditions

**Figure 3:**
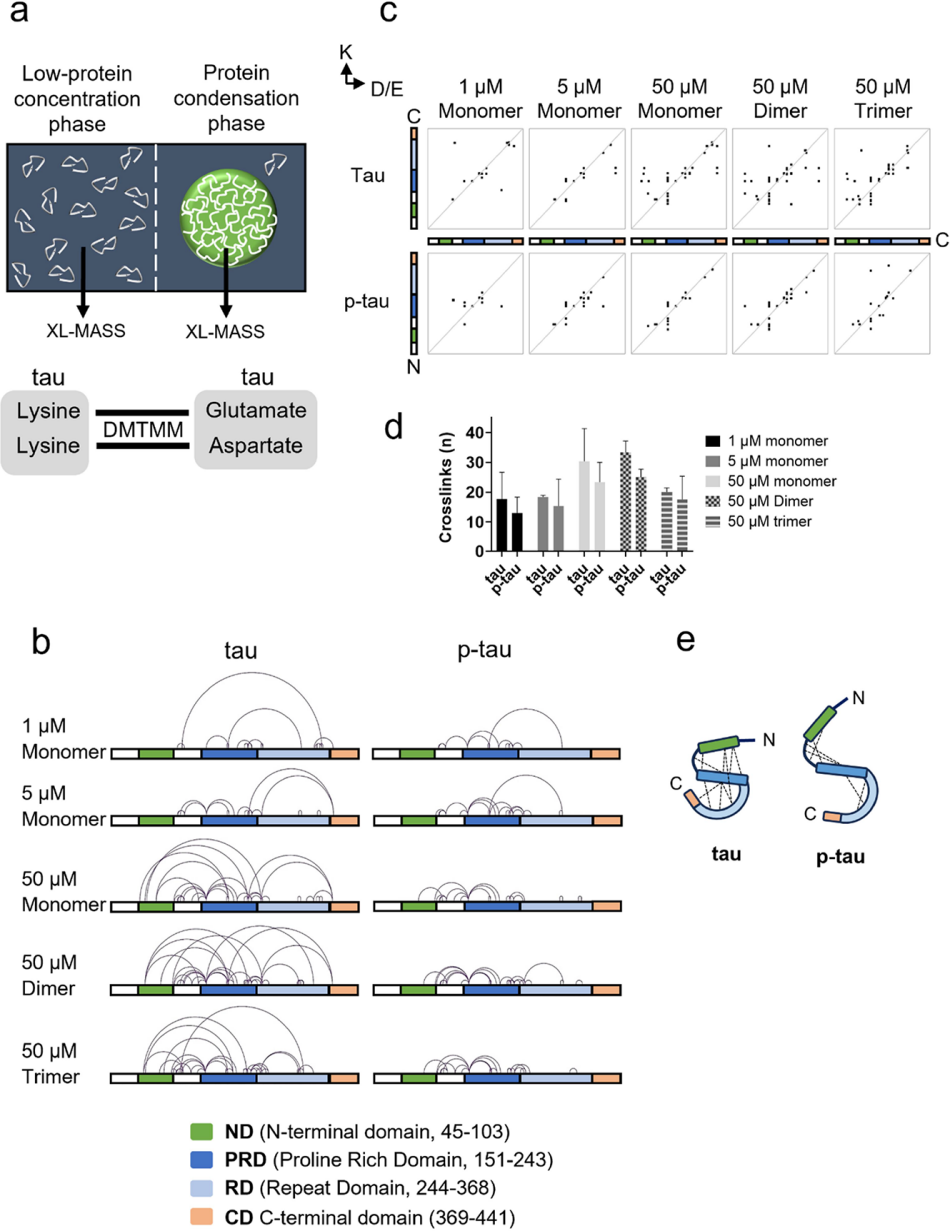
Conformational comparison of tau and p-tau by chemical cross-linking. (a) Schematic presentation of tau/p-tau in the solution containing 20 mM NaCl and 20 mM MOPS (pH 7.4) being cross-linked by DMTMM. (b) Identified cross-link connections. (c) Comparison of the cross-linking patterns of tau and p-tau using 2D contact maps. (d) Bar plot showing the number of cross-links in tau and p-tau (*N* = 3). (e) Schematic illustration of the proposed conformations of tau and p-tau under LLPS conditions, with cross-links indicated by dashed lines.

In this study, we investigated the conformational dynamics of tau and p-tau under LLPS conditions with the aim of elucidating their roles in the aggregation processes. Determining the conformation of IDPs in phase-separated states presents challenges due to their unstructured nature. To circumvent this problem, cross-linking mass spectrometry (XL-MS) was used to identify nearby amino acids and to elucidate protein conformation in different states. Because tau has an uneven charge distribution, we used 4-(4,6-dimethoxy-1,3,5-triazin-2-yl)-4-methylmorpholinium chloride (DMTMM) as the cross-linker, which is connected with the amine group of lysine (K) on one side and the carboxylic group of aspartate (D) or glutamate (E) on the other side (Figure 3a).

Using this technique, we investigated alterations in tau and p-tau topologies at three protein concentrations (1, 5, and 50 μM) because p-tau showed different LLPS propensities at these concentrations: no droplets at 1 μM, several small droplets at 5 μM, and numerous droplets at 50 μM (Figure S4). In our study, more cross-links were detected with increasing protein concentration (Figure 3b,c). Compared to p-tau, tau exhibited longer-range cross-links, particularly between ND and PRD and between PRD and RD, although the overall number of detected links did not significantly differ between the two proteins (Figure 3d). This indicates that tau adopts a more closed conformation, while the negative charges introduced by the phosphate groups in PRD (Figure 1e) interfere with the electrostatic interactions among these domains and render p-tau more extended and flexible (Figure 3e). The flexible p-tau is prone to form intermolecular interaction and coacervate to show LLPS. This property may also facilitate nucleation or seeding of aggregation, providing a mechanistic link between phosphorylation-driven conformational changes and pathological protein aggregation.

### RNA- and Phosphorylation-Induced Distinct Protein Coacervation Dynamics

**Figure 4:**
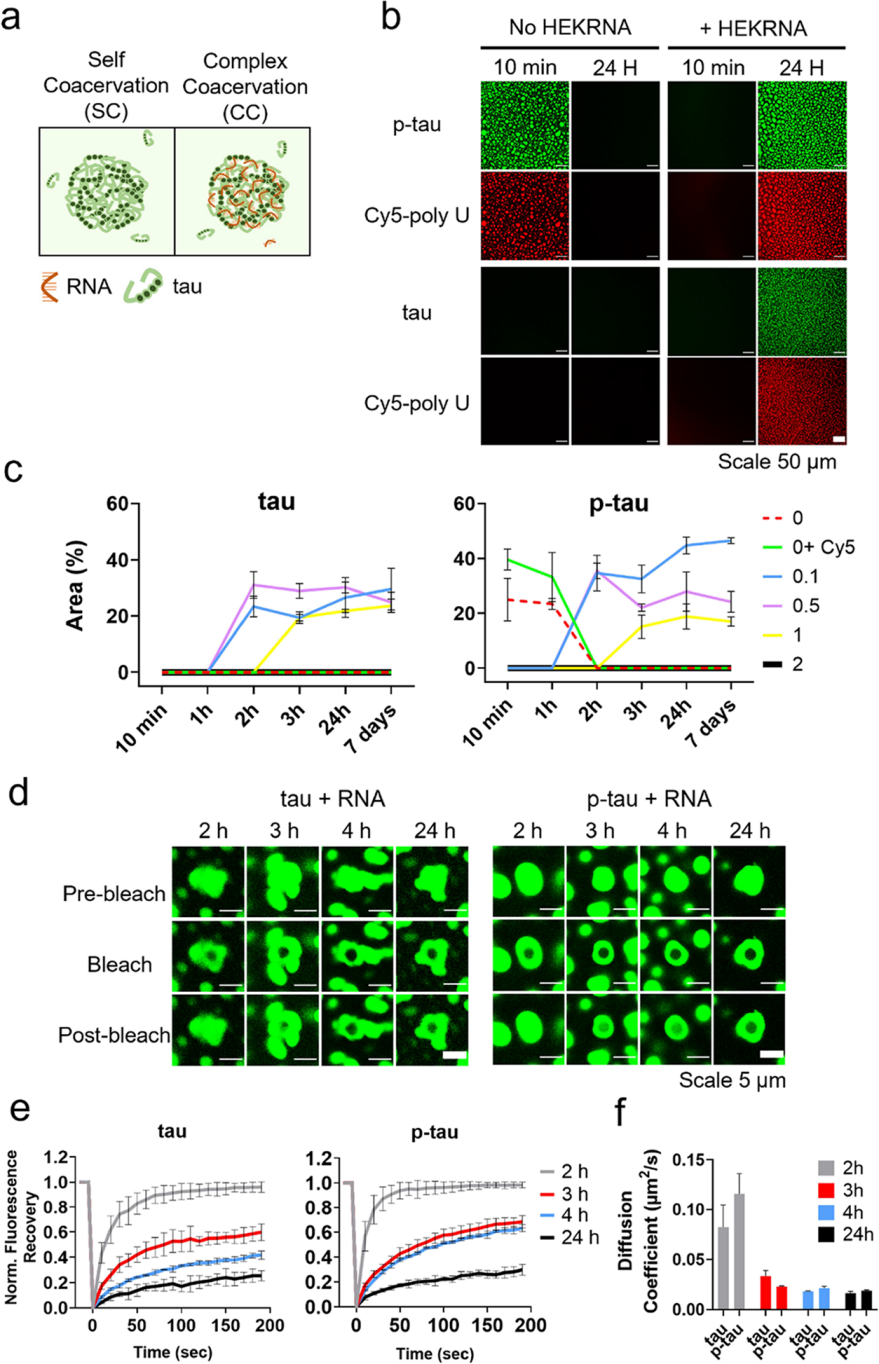
Distinct timeframes of SC and CC. (a) Schematic illustration of SC and CC. (b) Fluorescent images of p-tau/tau (50 μM, 10 μg) in the LLPS buffer with or without HEKRNA (RNA: protein = 1:10 by weight). AF488-labeled p-tau/tau proteins were mixed with unlabeled proteins in a 1:100 molar ratio. Cy5-labeled Poly U (25 nt) was added to trace RNA in a 1:500 ratio to HEKRNA. Images show tau/p-tau condensation properties without RNA and with a 1:10 HEKRNA-to-protein ratio. (c) Quantification of the area occupied by SC and CC droplets over a 7-day period. Values represent the mean ± SD from three independent samples. Red: no HEKRNA; green: no RNA, but Cy5-labeled Poly U added; blue, purple, yellow, and black: HEKRNA/protein ratio (w/w) of 0.1, 0.5, 1, and 2, respectively. (d) Comparison of protein fluidity inside HEKRNA-induced tau/p-tau droplets (RNA/protein ratio of 0.5). FRAP assay was conducted 2, 3, 4, and 24 h after incubation. Fluorescent images before bleaching, immediately after bleaching, and 200 s after bleaching are shown. (e) Time-dependent fluorescence recovery percentage of HEKRNA-induced tau/p-tau CC droplets after different incubation times. Images were taken every 10 s after bleaching. The fluorescence intensity in the bleached area was measured and averaged from three experiments. (f) Diffusion coefficients calculated from the data in (e).

We showed that phosphorylation initiates the SC of tau, which involves a demixing process driven by interactions between oppositely charged regions of the protein. In contrast, the introduction of RNA induces CC, wherein external negatively charged RNA facilitates further phase separation^4,5,26,30^ (Figure 4a). Tau can be found in both the cytosol and nucleus of the cells, although its function in the nucleus is not well-characterized.^64^ Moreover, tau binds to both RNA and DNA within the cell.^65^ The left lanes in Figure S5 prove that both tau and p-tau are free of nucleic acid. They can bind with RNA extracted from HEK293 cells, with an RNA-to-protein weight ratio of 1:1.5, as demonstrated by the electrophoretic mobility shift assay (EMSA) (Figure S5).

Furthermore, SC- and RNA-assisted CC occurred at different timeframes (Figure 4b). RNA extracted from HEK293 cells (HEKRNA) and tau/p-tau were incubated at different RNA-to-protein ratios. In all conditions, the protein concentration was maintained at 50 μM (10 μg), while the HEKRNA amount varied from 0 to 1, 5, 10, and 20 μg. The SC of p-tau occurred within 10 min but did not persist beyond 2 h (red and green lines in Figure 4c right). The tau protein did not exhibit LLPS without RNA (red and green lines in Figure 4c on the left). When RNA was added (at RNA-to-protein ratios of 0.1, 0.5, and 1), the CC for both p-tau and tau occurred with a delay of 1–2 h. These findings suggest that the spontaneous SC of p-tau followed a mechanism different from the RNA-assisted CC of tau and p-tau. While phosphorylation alone led to transient droplet formation, RNA interaction prolonged the stability and altered the internal mobility of the protein condensates—hinting at a transition toward a gel-like state, which might have escalated the aggregation over time. Additionally, poly A (40 nt; Invitrogen) exhibited the same CC-induction effect as HEKRNA (Figure S6). However, its CC-inhibition effect was weaker than HEKRNA, probably due to its short length. The same excitatory effect in low concentrations of RNA and inhibitory effect in higher concentrations of RNA were also observed in other RNA-binding proteins such as FUS and TDP43.^66,67^ Our data suggested that tau’s purity is important for observing its LLPS phenomenon. Different levels of RNA contamination might lead to controversial conclusions.

**Figure 5:**
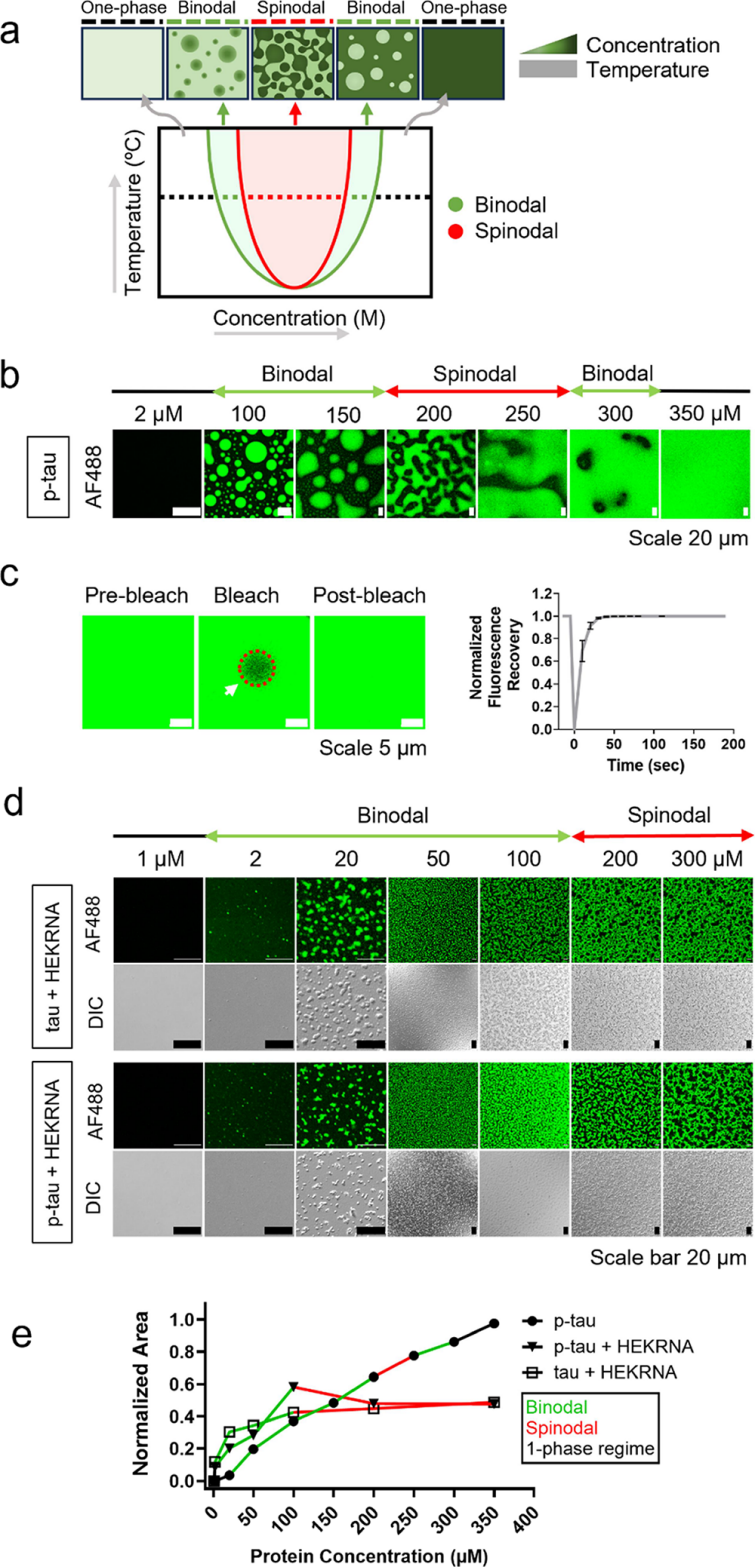
Concentration-dependent phase transition of p-tau SC and RNA-induced CC of tau/p-tau. (a) Schematic diagram illustrating concentration-dependent condensation. (b) Fluorescence images of various concentrations of p-tau, mixed with AF488-labeled p-tau (labeled:unlabeled = 1:100) in the LLPS buffer. (c) FRAP assay performed for the one-phase regime at a high p-tau concentration (350 μM). (d) Fluorescence and differential interference contrast (DIC) images of tau and p-tau at various protein concentrations. The proteins were mixed with corresponding AF488-labeled proteins (labeled:unlabeled = 1:100) and HEKRNA (RNA:protein = 0.5:1) in the LLPS buffer. (e) Droplet coverage area of p-tau SC and the CC of tau/p-tau with HEKRNA at various protein concentrations.

RNA in CC by binding and overlaying with the protein potentially facilitates more protein–protein interactions and hence triggers protein aggregation. Note that when RNase A was introduced immediately after the protein was mixed with RNA, no p-tau CC was observed after 2 h. When RNase A was added after mature droplet formation (2 h after p-tau was mixed with HEKRNA), most of the droplets disappeared, suggesting that RNase A can degrade RNA inside the droplets and destabilize the droplets. Only a few small droplets with irregular shapes, possibly the RNase-resistant p-tau aggregation cores, remained in the solution (Figure S7). In contrast, the p-tau SC was not affected by RNase A.

In the RNA-induced CC of tau and p-tau, a marked decrease in protein mobility within the droplets was observed over time. As shown by the FRAP test, the later photobleaching was conducted, the slower the fluorescence recovery rate was observed (Figure 4d,e). Compared to the diffusion coefficient of 0.144 μm^2^/s ± 0.022 obtained from p-tau spontaneous SC, as shown in Figure 2i, the diffusion coefficients for RNA-induced CC were slower: 0.082 ± 0.022 for tau and 0.116 ± 0.020 μm^2^/s for p-tau after 2 h of incubation with RNA. Additionally, the diffusion coefficients for both tau and p-tau dropped sharply when incubation was extended to three or more hours (Figure 4f). Furthermore, the final recovery percentage after 200 s also decreased with longer coacervation incubation times. When RNA-induced CC droplets of tau and p-tau after 2 h incubation achieved full recovery, similar to p-tau’s self-coacervation, the recovery percentage decreased to around 20% for both tau and p-tau when the incubation was extended to 24 h.

Fluorescence images indicated that phosphorylation-induced SC and RNA-induced CC occurred at different timeframes. SC formed immediately but remained there for no more than 2 h. CC formed after 2 h but did not disassemble during our observation period (7 days). FRAP data showed p-tau in the SC droplets had high flexibility (Figure 2i) but low stability (Figure 4c), while the flexibility of tau/p-tau in the RNA-induced CC droplets decreased with time (Figure 4e).

### Transition Pattern from Nucleation to Demixing of Tau/P-tau: Difference between SC and CC

To investigate the LLPS properties of tau/p-tau, we analyzed both demixing stabilities (binodal phase separation) and local phase instability (spinodal phase separation). As illustrated in Figure 5a, increasing the protein concentration at a constant temperature promotes protein condensation. However, this condensation is transient, as the rising molecular density eventually leads to spinodal decomposition.^5,68^

The sequence of transitions during p-tau SC is depicted in Figure 5b. No LLPS was detected at concentrations below 5 μM. However, at concentrations between 5 and 150 μM, clear binodal phase separation was observed.^68,69^ Note that as the concentration increased from 200 to 250 μM, we observed an instability in the droplet shape, known as spinodal decomposition. At a constant room temperature, further increases in protein concentration led to changes in the spatial distribution of the condensed area, transitioning through the binodal and spinodal phases and eventually returning to a one-phase mixed regime. A FRAP assay conducted in the one-phase regime at very high p-tau concentrations showed complete recovery within tens of seconds after photobleaching (Figure 5c), with the diffusion coefficient calculated as 0.134 ± 0.021 μm^2^/s. This complete recovery after photobleaching suggests that even at high protein concentrations molecules dynamically move within the condensed phase.

Unlike p-tau SC, increasing the protein concentration in tau/RNA and p-tau/RNA mixtures led to a transition of droplets solely from a stable two-phase state (binodal) to an unstable spinodal state. For both tau and p-tau mixed with HEKRNA, no LLPS was observed at concentrations below 2 μM. However, concentrations between 2 and 100 μM exhibited a nucleation binodal pattern, while those above 100 μM transitioned to a spinodal state (Figure 5d). Regardless of whether tau or p-tau was used, the protein/RNA mixture remained in the spinodal state, even with further increases in the protein concentration (Figure 5e). Our findings demonstrated that the phase behaviors of tau and p-tau were intricately linked to their interactions with RNA, affecting both the dynamics of movement and the morphology of protein condensates. In p-tau SC, the spinodal state was a transitional phase between two binodal phases, whereas in RNA-induced CC, the spinodal state exhibited a stable shape, even at the highest protein concentration. This stabilization indicated the formation of a gel-like state when the protein was treated with RNA.

### Driving Force for the Formation of SC and CC of P-tau

The quest to unveil the intricate mechanisms steering LLPS remains a subject of ongoing debate.^29,58^ To probe into the mechanisms underlying tau coacervation, we examined the contributions of electrostatic and hydrophobic interactions in the self-coacervates and RNA-induced complex coacervates of p-tau as well as the effects of pH on tau and p-tau LLPS.

**Figure 6:**
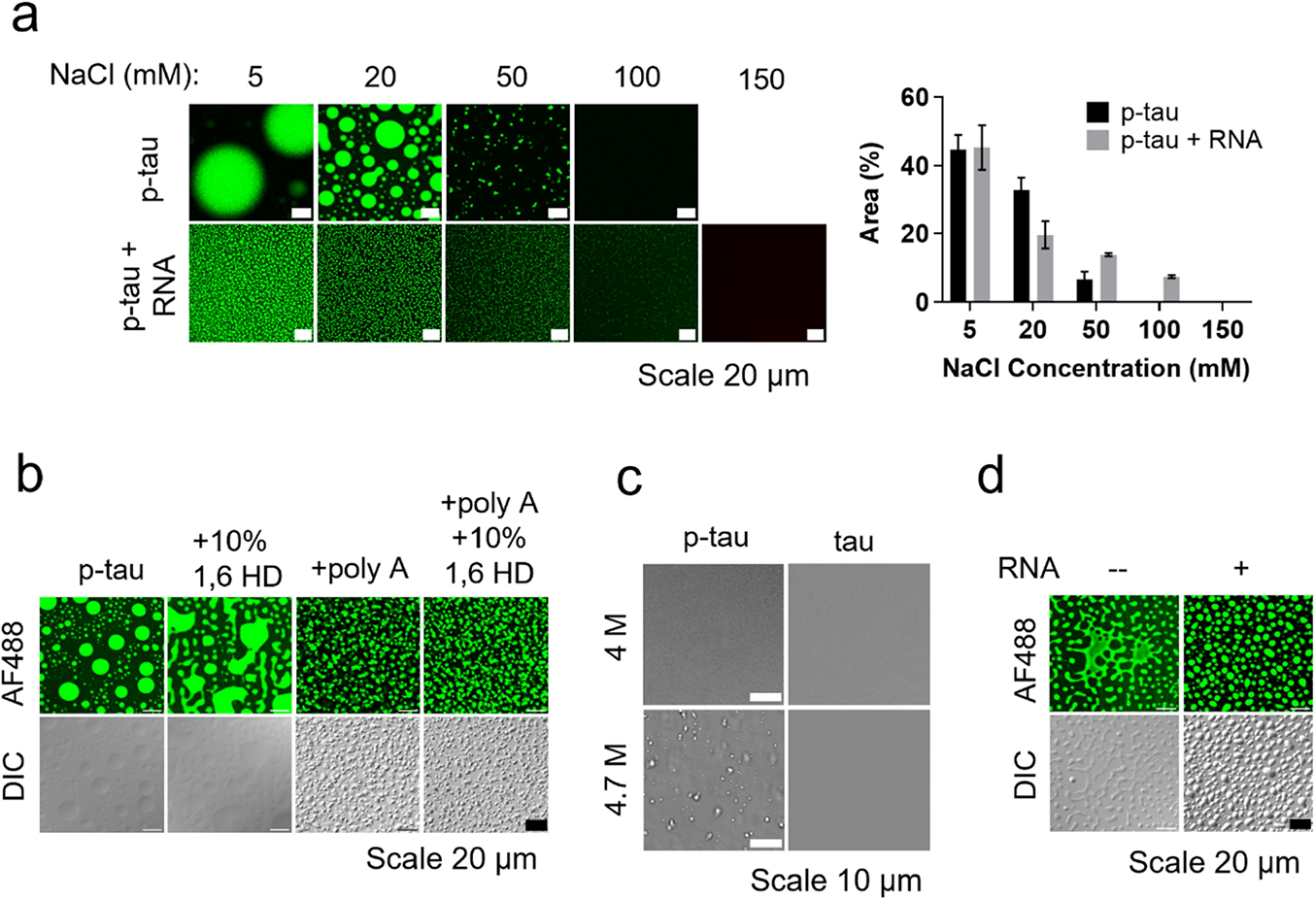
Driving forces involved in p-tau SC and CC. (a) Fluorescence images of p-tau with and without RNA at different salt concentrations. p-tau (50 μM) and AF488-labeled p-tau (0.5 μM) were dissolved in 20 mM Tris (pH 7.4) with NaCl concentration of 5–150 mM and HEKRNA (RNA:protein = 0.5:1 by weight). The area covered by the droplets was quantitated on the right side of the images. (b) Fluorescence and DIC images of p-tau (50 μM, 10 μg) and AF488-labeled p-tau (0.5 μM) with or without poly A (1 μg) and 10% 1,6-HD in the LLPS buffer. (c) DIC images of 50 μM p-tau and tau in a buffer containing 20 mM Tris (pH 7.4) and 4 or 4.7 M NaCl. (d) Fluorescence and DIC images of p-tau (50 μM) and AF488-labeled p-tau (0.5 μM) with or without HEKRNA in a solution containing 20 mM NaCl and 20 mM Tris at pH 4.5.

First, we noticed that the droplet sizes of CC formed by p-tau/HEKRNA were notably smaller than those of p-tau’s self-coacervate self-catalysts (Figure 6a). As the NaCl concentration increased, the droplet sizes in both scenarios of condensation decreased. This suggests that the strength of electrostatic interactions, which diminishes with the rising salt concentration, plays a crucial role in driving both the SC and CC processes.

Second, we used 1,6-hexanediol (1,6-HD), a disruptor of hydrophobic interaction, to examine the impact of hydrophobic interaction on p-tau LLPS. 1,6-HD significantly destabilized p-tau’s SC droplets and promoted p-tau to change from the binodal state into the spinodal state but did not affect p-tau’s RNA-induced droplets (Figure 6b). These data suggested that the hydrophobic interaction was important for SC but not for CC. Moreover, extremely high concentrations of NaCl (4.7 M) led to p-tau condensation through dehydration. In contrast, the same salt concentration failed to induce the droplet formation of tau (Figure 6c). This result is different from the previous report^5^ in which tau in a buffer containing 4.7 M NaCl underwent SC without any crowding agent.

It has been reported that tau can accumulate and spread between neurons via extracellular vesicles, which have a lower pH.^70,71^ To explore the pH effect, we examined p-tau’s SC and CC at an acidic condition (Figure 6d). At pH 4.5, p-tau’s SC displayed altered behavior, adopting a more spinodal shape, while CC remained unaffected.

### Fibrillization of Tau/P-tau Influenced by Different Inducers In Vitro

**Figure 7:**
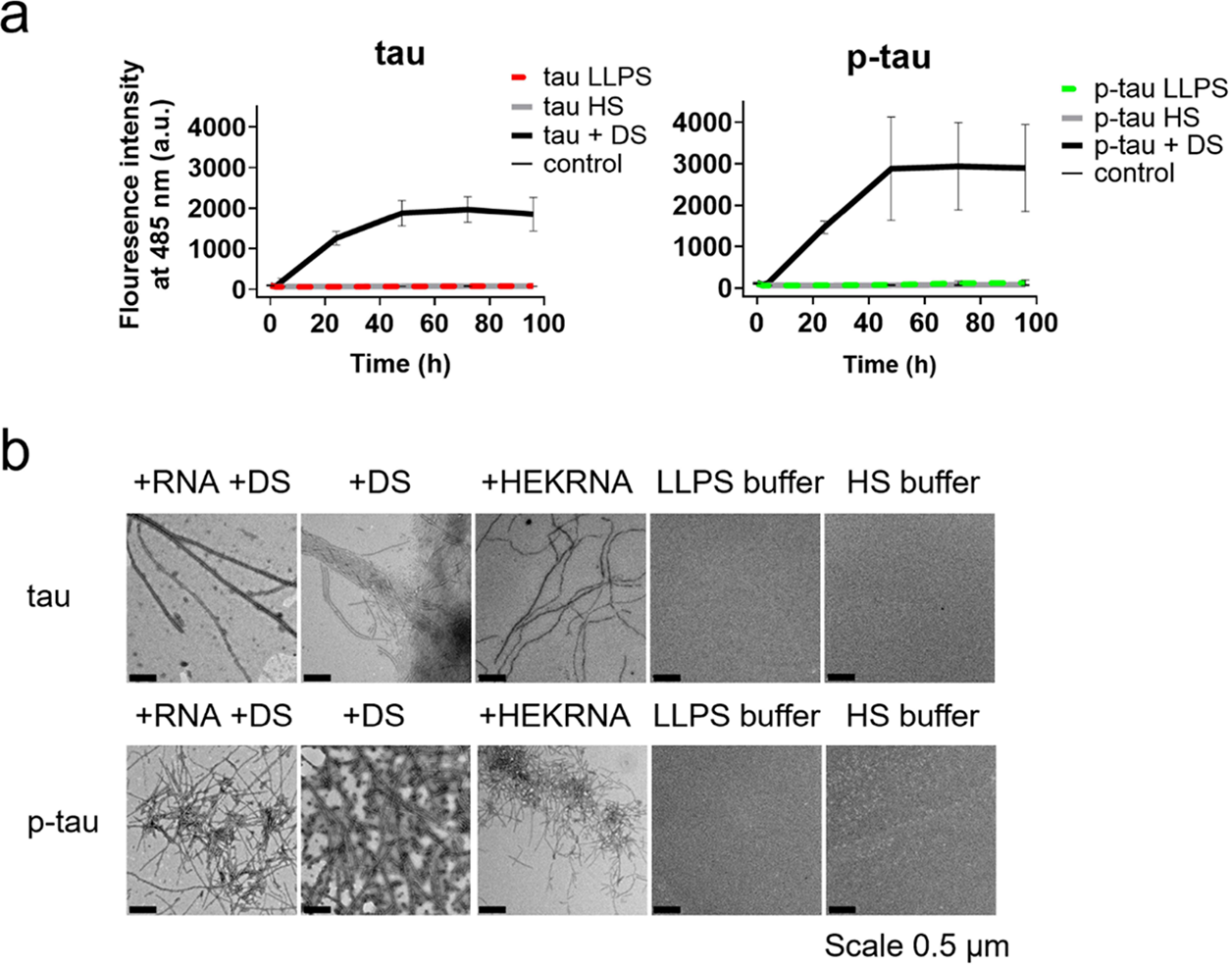
Fibrillization of tau and p-tau by different inducers. Tau and p-tau (20 μM) were incubated in the LLPS buffer (20 mM NaCl, 20 mM Tris, pH 7.4), in the HS buffer (150 mM NaCl, 20 mM Tris, pH 7.4), in the LLPS buffer containing HEKRNA (RNA:protein = 0.5:1 by weight), and in the LLPS buffer containing 40 μg/mL DS. (a) Fibrillization kinetics were monitored by using the ThT binding assay except for the RNA-containing samples. DS in the LLPS buffer was used as the control. (b) TEM images of tau and p-tau incubated with different inducers.

Phosphorylation is a crucial post-translational modification that regulates numerous cellular processes, including protein aggregation.^18,72^ However, the direct relationship between phosphorylation and aggregation of tau remains elusive.^17^ In some studies, phosphorylation promoted p-tau aggregation and the formation of inducer-free p-tau fibrils.^24,48^ Here we explored whether p-tau undergoing LLPS effectively promotes fibrillization as the high protein density within droplets may serve as a prerequisite for protein association or fibrillization.^1,26^ To test this in vitro, tau and p-tau were incubated in the LLPS buffer at pH 7.4, accompanied by 10 μM thioflavin T (ThT), to monitor fibrillization. Fibrillization was also tested at a higher salt concentration (150 mM NaCl), where condensation could not occur. Another tested condition involved proteins with HEKRNA, providing an opportunity to investigate the effect of CC on tau/p-tau fibrillization in vitro. Additionally, proteins incubated with and without dextran sulfate (DS) were tested, as DS has been shown to induce a structure resembling the fibrils found in AD brains, as evidenced by cryo-electron microscopy.^73^ Because RNA has a high affinity for ThT, we did not monitor the fibrillization of the RNA-containing samples using the ThT binding assay. Figure 7a shows that only DS-induced proteins exhibited a significant increase in the ThT fluorescence signal and that p-tau had a higher intensity than tau. The fibrillization results were validated using TEM; it was found that fibrillization can be induced by DS or HEKRNA (Figure 7b). Notably, in the presence of HEKRNA, both tau and p-tau formed stable liquid droplets at room temperature (Figure 5d). However, upon incubation with shaking at 37 °C, they transitioned into fibrils (Figure 7b). It suggests that shaking might act as a factor redirecting the protein aggregation pathway. In contrast, p-tau in the LLPS buffer, under the same incubation conditions but without RNA addition, did not form fibrils even at a protein concentration as high as 100 μM. This further suggests that the LLPS state is not an intermediate on the fibrillization pathway (Figure S8).

**Figure 8:**
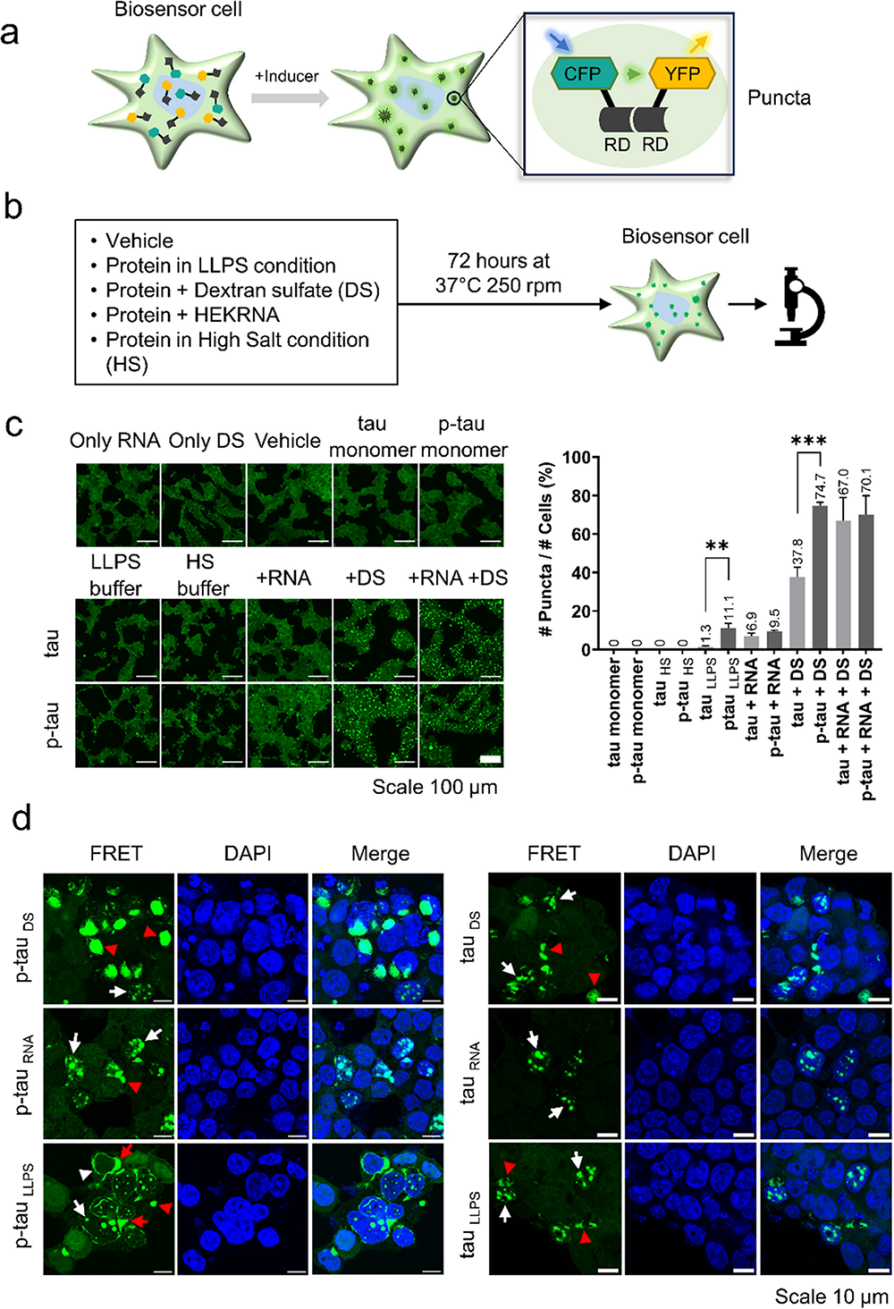
In-cell tauRD association induced by tau/p-tau in different states. (a) Schematic figure illustrating the enhanced association of the repeat domain (RD) of tau in biosensor cells following treatment with inducers. In these cells, the FRET signal is activated through the association of tau RD P301S-CFP (donor) and tau RD P301S-YFP (acceptor). (b) Tau and p-tau were dissolved in either the LLPS buffer (with or without inducers such as DS or RNA) or the HS buffer and incubated at 37 °C with shaking for 3 days. Subsequently, tau and p-tau in various states were added to the cell medium to a final protein concentration of 1 μM, and the culture was incubated for an additional day. (c) Fluorescence imaging of cells treated with tau/p-tau in different states. For the monomer groups, tau and p-tau were dissolved in the LLPS buffer and added to the cell culture immediately. The normalized percentage of puncta formation was quantified and is shown on the right side of the images as mean ± SD (*n* = 3). Statistical analysis between the tau and p-tau samples was performed using one-way ANOVA, with significance levels indicated as **P < 0.01 and ***P < 0.001. (d) The locations of tauRD in cells incubated with DS-treated p-tau or tau (p-tau_DS_ or tau_DS_), RNA-treated p-tau or tau (p-tau_RNA_ or tau_RNA_), and p-tau or tau treated in LLPS condition (p-tau_LLPS_ or tau_LLPS_) were compared. p-tau_DS_ and tau_DS_ induced amorphous accumulation in the cytosol (AMO, red arrowhead) as well as aggregation inside the nucleus (NUC, white arrow). p-tau_RNA_ and tau_RNA_ primarily appeared as speckles inside the nucleus (NUC, white arrow). However, p-tau_LLPS_ induced accumulation primarily at the nuclear envelope (NE, white arrowhead) or in close proximity (∼1.5 μm) to the nucleus (CYT, red arrow). In contrast, only NUC and AMO can be found in the tau_LLPS_ group.

### In-Cell TauRD Association Induced by Tau/P-Tau in Different States

Tau aggregates can promote tau association in the biosensor cells that stably express the tau R domain (tauRD) fused with a CFP/YFP-FRET reporter (Figure 8a).^74^ To see how tau and p-tau condensates affected tauRD aggregation in the biosensor cells, different tau and p-tau samples shown in Figure 7b were added to the medium of the biosensor cells, along with lipofectamine for facilitating cell membrane penetration (Figure 8b). After 24 h, the cells were assessed for fluorescence puncta formation. According to the results, tau/p-tau treated with DS (tau_DS_ or p-tau_DS_) caused the most pronounced puncta formation in the cells (Figure 8c). Tau/p-tau treated with RNA (tau_RNA_ or p-tau_RNA_) also induced punctation formation, albeit to a lesser extent. Note that significant puncta formation was observed in cells treated with p-tau under the LLPS condition (p-tau_LLPS_), whereas very few puncta were found in the tau_LLPS_ sample. Neither tau/p-tau monomer nor tau/p-tau in the HS buffer induced puncta formation in the cell.

TauRD associations within cells can be categorized into several types based on their cellular location and fluorescent intensity: (1) large, bright amorphous accumulations in the cytosol detached from the nucleus (AMO), (2) bright cytoplasmic inclusions approximately 1.5 μm in radius around the nucleus (CYT), (3) smaller nucleus accumulations (NUC), and (4) a dimmer, nonuniform thin layer at the nuclear envelope (NE).^74^ Hochmair et al. have shown tau accumulation at the NE in AD brains.^74^ Additionally, PEG-induced tau LLPS was found to lead to punctal formation in cells, whereas tau alone did not induce puncta.

In addition to the number of puncta, we also examined where these tau- and p-tau-induced FRET puncta localized in cells. Our results clearly demonstrated that tau and p-tau, in different states, induce tauRD aggregation in distinct cellular locations (Figure 8d). Although both DS and RNA can induce fibrillization, numerous AMO structures were found in cells treated with p-tau_DS_ and tau_DS_ (Movies S3 and S4), while NUC was the predominant type of aggregation observed in cells treated with p-tau_RNA_ and tau_RNA_ (Movies S5 and S6). In particular, the NE of tauRD was exclusively observed in cells treated with p-tau_LLPS_ (Movie S7). Only a few puncta were found in cells treated with tau_LLPS_, in either the cytosol (AMO) or the nucleus (NUC) (Movie S8).

Although p-tau droplets disappeared after 2 h incubation and tau did not form LLPS (Figure 4b), it is possible that some “seeds” formed during the 72 h incubation of tau and p-tau in the LLPS state. This is evidenced by tau_LLPS_ and p-tau_LLPS_ inducing tauRD aggregation in biosensor cells, whereas tau and p-tau monomers did not. The distinct intracellular puncta locations observed suggest that the “seeds” triggering tauRD aggregation for tau_LLPS_ and p-tau_LLPS_ likely differ. These seeds may be either very small or present in low quantities that we cannot detect by now. Our findings suggest the formation of an intermediate during LLPS that could play a pivotal role in the aggregation process and warrant further investigation.

## Conclusions

Our study underscored the intricate relationships among phosphorylation, LLPS, and protein aggregation. Phosphorylation emerged as a crucial modulator of LLPS, significantly influencing tau’s biophysical properties and its aggregation potential. In particular, p-tau with 2–9 phosphate groups displayed an enhanced propensity for LLPS, forming dynamic, temperature-sensitive droplets with reversible behavior. This suggests that phosphorylation indirectly promotes aggregation by facilitating LLPS, creating a favorable environment for nucleation and protein–protein interactions. The structural differences observed between tau and p-tau under LLPS conditions, with p-tau adopting a more exposed conformation, indicate that LLPS-mediated conformational changes play a critical role in aggregation processes.

Our findings also highlighted the distinct mechanisms and timeframes governing phosphorylation-induced SC and RNA-induced CC of tau. RNA significantly impacted the stability and morphology of tau condensates, driving their transition toward a gel-like state, which exacerbated aggregation over time. The sensitivity of p-tau to electrostatic and hydrophobic interactions under varying ionic and pH conditions further underscores the complexity of LLPS dynamics. Notably, RNA-induced LLPS exhibited distinct phase behavior compared to phosphorylation-induced LLPS, highlighting the importance of RNA in modulating tau’s condensation and aggregation tendencies within cellular environments. Furthermore, p-tau SC can induce tauRD aggregation at the nuclear envelope, as observed in AD brain sections, implying that p-tau SC might play a crucial role in AD pathogenesis.

In conclusion, our study advances the understanding of how phosphorylation and RNA interactions modulate tau’s LLPS and aggregation dynamics, underlining the importance of these molecular mechanisms in the context of neurodegenerative diseases, particularly tauopathies.

## Materials and Methods

### Expression and Purification

Two constructs, pMK1013-GSK3β-tau-1N4R and pMK1013-tau-1N4R, were used to generate p-tau and tau, respectively, both of which are part of the previously proposed PIMAX system.^48^ These two plasmids were transformed into BL21-CodonPlus (DE3)-RIPL competent cells. The pMK1013-GSK3β-tau-1N4R construct expresses two proteins: His6 tag-Fos-thrombin-GSK3β and Jun-TEV-tau, while pMK1013-tau-1N4R expresses the corresponding proteins without GSK3β. The PIMAX system uses Fos and Jun leucine zipper to facilitate the interaction between tau and GSK3β.^48^ After plasmid transformation, a single bacterial colony was inoculated into the LB medium containing 100 μg/mL ampicillin. Following overnight incubation, the culture was transferred to 1 L of LB medium supplemented with 2 mM MgSO_4_ and 100 μg/mL of ampicillin. The cells were then incubated at 37 °C with vigorous shaking at 250 rpm. When the optical density (OD) reached between 0.6 and 0.8, isopropyl thiogalactopyranoside was added to a final concentration of 0.1 mM to induce protein expression. The harvested bacterial cells were then lysed in 10 mL of lysis buffer containing 20 mM Tris (pH 6.0), 100 mM NaCl, 1 mM phenylmethanesulfonylfluoride, 1 mg/mL lysozyme, 0.2 mM orthovanadate, and a Roche protease inhibitor mini-tablet. The mixture was incubated at 30 °C for 0.5 h, with gentle inversion every 10 min. The cells were then disrupted using sonication at 30% amplitude. Following sonication, the mixture was centrifuged at 17,000*g* for 40 min at 4 °C to remove cellular debris. Tau protein is remarkably resistant to heat and acid treatment without losing its functionality. Therefore, the lysate was subsequently subjected to a boiling water bath to denature the other proteins, leaving the tau protein intact. To remove aggregated proteins, the mixture was centrifuged at 20,000*g* for 45 min at 4 °C. Next, TEV protease was added at a ratio of 1:100 (TEV:tau) to cleave tau/p-tau from the fusion protein. After TEV cleavage, the sample was loaded onto a strong ion-exchange chromatography column (SP-sepharose, HiPrep SP Fast Flow 16/10). Fractions containing the desired protein were collected and further purified using a size exclusion column (Superdex 200 Increase 10/300 GL). Fractions containing tau/p-tau proteins were pooled, and the solution was concentrated using an Amicon 3 kDa spin column. The protein concentration was determined using a bicinchoninic acid assay, and the purified protein was stored at −80 °C.

### Fibrillization Assay and Fibril Visualization

Tau and p-tau proteins were incubated in a buffer containing 10 μM ThT, 20 mM NaCl, and 20 mM Tris (pH 7.4) for 72 h at 37 °C. This incubation process was carried out with continuous orbital rotation using a Gemini EM microplate reader (Molecular Devices, USA). Upon binding to the hydrophobic pockets within amyloid fibrils, the ThT dye exhibited a significant enhancement in the fluorescence quantum yield, resulting in a robust emission peak at 485 nm when excited at 440 nm. To assess the fibril morphology, TEM was employed. A 5 μL sample was deposited onto carbon-coated 300-mesh copper grids and allowed to adsorb for 3 min. The grid was then washed with 10 μL of deionized water to remove excess salt. A 30-s staining process using 2% phosphotungstic acid (PTA) was applied. After being dried overnight, the grids were examined using an FEI Tecnai G2 F20 S-TWIN electron microscope.

### Detection of Protein Sequences and Modifications by LC–MS/MS

The proteins were run on a 15% SDS-PAGE gel; the band corresponding to the protein of interest was excised, and it was destained with 25% acetonitrile and 25 mM ammonium bicarbonate. The samples were reduced by using 10 mM dithioerythreitol (DTT), 8 M urea, and 25 mM ammonium bicarbonate (pH 8.5) at 37 °C for 1 h. Subsequently, the samples were alkylated with 25 mM iodoacetamide in 25 mM ammonium bicarbonate at pH 8.5 in the dark at room temperature for 1 h. The reaction was quenched with the addition of 25 mM DTT in 25 mM ammonium bicarbonate.

For digestion, the samples were treated with MS-grade Lys-C at an enzyme-to-protein ratio of 1:50 in 25 mM ammonium bicarbonate (pH 8.5) at 37 °C for 3 h, ensuring that the final urea concentration was less than 4 M. After Lys-C digestion, an equal amount of sequencing-grade trypsin in 25 mM ammonium bicarbonate (pH 8.5) was added, and the mixture was further reacted at 37 °C for 16 h, with the final urea concentration maintained below 1 M. The digestion reaction was quenched by adding 0.1% formic acid, and the solution was dried using Speedvac. The resulting peptide mixture was aliquoted, desalted, and concentrated using a C18-ZipTip (Millipore). Finally, elution was performed using a solution containing 50% acetonitrile and 0.1% formic acid.

NanoLC–nanoESI-MS/MS analysis was conducted by using a nanoAcquity system (Waters, Milford, MA) coupled with an LTQ Orbitrap Velos hybrid mass spectrometer (Thermo Electron, Bremen, Germany) equipped with a Nanospray Flex interface. Peptide mixtures were loaded onto a 75-μm internal diameter, 25 cm long C18 BEH column (Waters, Milford, MA) packed with 1.7-μm particles with a pore size of 130 Å. Solvent A (0.1% formic acid in water) is used as the starting solvent for the gradient separation of the peptides on the C18 column. As the gradient progresses, solvent B (acetonitrile with 0.1% formic acid) increases and the ratio of solvent A decreases. These peptide mixtures were then separated using a segmented gradient: from 5 to 25% over 27.5 min and from 25 to 35% over 2.5 min with solvent B. The flow rate was set to 300 nL/min, and the column temperature was maintained at 35 °C.

The mass spectrometer operated in the data-dependent mode, where the initial survey full-scan MS spectra were obtained using an orbitrap (*m*/*z* 350–1600). The resolution was set to 60,000 at *m*/*z* 400, and the automatic gain control target was set to 10^6^. The 10 most intense ions were selected for higher-energy collisional dissociation (HCD) MS/MS fragmentation and analysis in the orbitrap. The selected ions were dynamically excluded for 60 s. During the MS/MS fragmentation and analysis, a resolution of 7500, an isolation window of 2 *m*/*z*, and a target value of 50,000 ions were used, with a maximum accumulation time of 250 ms. Fragmentation was achieved by applying a normalized collision energy of 35% and an activation time of 0.1 ms. Ions with unrecognized or singly charged states were excluded.

Data were analyzed using Mascot software against the NCBIport and Swissport databases, allowing trypsin cleavage and a 10-ppm mass tolerance. Modifications were specified for tyrosine, threonine, and serine phosphorylation, and identifications were filtered using a false discovery rate (FDR) threshold of <0.02.

### Mass Photometry

Data for tau and p-tau were obtained using a TwoMP instrument (Refeyn, UK). Both proteins were measured at a concentration of 2.5 nM. A 20 μL sample of the diluted tau and p-tau solutions was placed in a gasket mounted on a clean coverslip slide. The data were analyzed by determining the peak mass and fitted to a normal distribution of particle counts, with σ representing the standard deviation of the Gaussian curve.

### Native Mass Spectrometry

The proteins were buffer-exchanged into 200 mM ammonium acetate buffer at pH 7.5. The prepared samples were then loaded into in-house-fabricated gold-coated emitters and sprayed into a modified Q-Exactive mass spectrometer (Thermo Fisher Scientific). The MS parameters were set as follows: spray voltage of 1.1 kV, capillary temperature of 200 °C, S-lens RF level of 200, and resolution setting of 12,500. To avoid collisional activation of the ions before transmission into the HCD cell, a gentle voltage gradient was applied, with voltages set as follows: injection flatapole at 5.0 V, inter flatapole at 4.0 V, bent flatapole at 2.0 V, and transfer multipole at 1.0 V. An HCD energy of 10 V was applied. Data were analyzed and processed manually using the Thermo Xcalibur Qual Browser (version 4.4.16.14). UniDec (Universal Deconvolution) software was used to quantify the relative abundance of tau variants with different numbers of phosphate groups.

### Phase Separation and Droplet Formation Detection

The evaluation of the visual turbidity and reversibility of the protein phase separation was conducted by observing light scattering at a wavelength (λ) of 400 nm using a UV/vis spectrophotometer equipped with a temperature control module (UV/vis spectrophotometer V630 Bio, JASCO). Changes in turbidity were recorded as the temperature transitioned from 4 °C to higher values at a rate of 1 °C min and vice versa.

Phase separation and subsequent droplet formation were detected using a DIC/Leica SP5 inverted confocal microscope at room temperature, operating with 40× optical magnification. In this study, both tau and p-tau were labeled with Alexa Fluor 488 (Invitrogen, A20000). To investigate the conjugation of the amine-reactive label with the proteins, 10 μL of dye in DMSO (10 mg/mL) was added to 100 μL of tau or p-tau to a final protein concentration of 100 μM. The labeling was performed in a buffer containing 20 mM Tris and 20 mM NaCl at pH 7.4 (LLPS buffer). Following overnight incubation at 4 °C, any unbound dye was removed using MicroSpin G-25 desalting columns (Cytiva, GE27-5325-01), with centrifugation at 1000*g*.

Dye labeling can influence protein behavior. To minimize this impact, fluorescently labeled protein to unlabeled protein at a ratio of 1:100 was used in the investigation of phase separation. The experimental setup involved combining the buffer, unlabeled protein (with concentration depending on the experiment), and labeled protein. This mixture was then mounted onto a glass microscope slide, covered with a coverslip, and sealed by using transparent nail polish to prevent edge evaporation. The prepared sample was examined with a confocal microscope. The partition coefficient (*K*) of the tau/p-tau droplets was calculated using the following formula.^75^
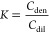
where *C*_den_ and *C*_dil_ indicate the dense phase and dilute phase coverage, respectively. These values were measured from the images by using ImageJ software.

### FRAP Analysis

The FRAP analysis, conducted using a Leica SP5 confocal microscope, demonstrated that the molecules were actively moving within the droplets. An ROI measuring two micrometers in diameter inside the droplet was subjected to laser photobleaching five times at 70% intensity, followed by recording the image every 10 s to prevent further photobleaching during imaging. Immediately after photobleaching, fluorescence recovery occurred due to the diffusion-driven interaction between the fluorescent molecules inside and outside the photobleached region. Time-lapse images captured the fluorescence recovery within this bleached area at 10-s intervals over a period of 3 min. The diffusion coefficient (*D*) was calculated using a 2D infinite model and the equation proposed by Axelrod and Soumpasis.^76,77^
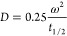
where *D*, *t*_1/2_, and ω represent the apparent diffusion coefficient, recovery half-time, and radius of the circular bleached area, respectively.

### EMSA

The binding interactions between various concentrations of the proteins and HEKRNA were observed after 1 h of incubation at room temperature. The resulting complexes were visualized using a 1% agarose gel containing SYBR Safe DNA gel stain (1/10,000, v/v) (Invitrogen, S-33102).

### TauRD P301S FRET Biosensor

The tauRD P301S FRET biosensor cells (CRL-3275) were purchased from ATCC.^78^ This cell line was engineered by introducing two separate lentivirus constructs carrying tauRD P301S-CFP and tauRD P301S-YFP into HEK293T cells. The addition of tau seeds to the culture medium of these biosensor cells triggered the aggregation of endogenous tau reporter proteins, resulting in a detectable FRET signal via flow cytometry and microscopy. For the experiments, the cells were cultured in a 12-well plate with 2 × 10^5^ cells suspended in 800 μL of DMEM (Thermo Fisher) supplemented with 10% FBS (Gibco), 1% GlutaMAX (Gibco), and 1% penicillin-streptomycin antibiotics (Gibco). The plate had poly-l-Lysine-coated (0.01%) glass coverslips. The cells were incubated overnight at 37 °C in a 5% CO_2_ atmosphere. When the cells reached 70–80% confluency, the seed solution (1 μM final concentration) was mixed with 3.5 μL of Lipofectamine 3000 (Invitrogen), then diluted to 160 μL using the Opti-MEM reduced serum medium, and left at room temperature for 15–20 min. The solution was then added to DMEM (without FBS/antibiotics) to reach a final volume of 800 μL. This mixture was introduced to each well of the 12-well plate and allowed to incubate at 37 °C for 24 h.^33^ Next, the cells were treated with a fixative agent, 4% paraformaldehyde, in 1X PBS for 15 min to form covalent cross-links among the molecules. Then, the cells were treated with Tween 20 for 10 min, which facilitated the formation of pores in the cell membrane without compromising its integrity. Following three washes, DAPI was employed as a counterstain, specifically targeting nucleic acids. The cells were then rinsed once with deionized water and ultimately mounted on a slide for microscopy. Tau aggregate formation within the cells was visualized using an Olympus FV3000RS inverted confocal fluorescence microscope with excitation by a CFP 445 nm laser and detection through a YFP 530/25 nm emission filter. The cells exhibiting this interaction were quantified by enumerating FRET-positive cells relative to the total cell count indicated by the DAPI-stained nuclei.

### Cross-Linking Assay

Tau and p-tau (1, 5, and 50 μM) were cross-linked with 40 mM DMTMM in a buffer containing 20 mM MOPS and 20 mM NaCl (pH 7.4) at 37 °C for 1 h. The reaction was stopped by adding 1 M Tris buffer to a final concentration of 50 mM. The cross-linked proteins were separated using SDS-PAGE. The cross-linked proteins moved faster and showed bands at lower positions on SDS-PAGE. The bands at higher positions in the high-concentration samples represented cross-linked dimers and trimers. The selected bands were excised for in-gel digestion. The proteins were reduced with dithioerythritol and then alkylated with iodoacetamide. The samples underwent sequential digestion, first with Lys-C protease at 37 °C for 3 h and then with trypsin at 37 °C overnight. After digestion, the resulting peptide mixtures were extracted and purified by using C18 ZipTips. The eluted peptides were then dried using a SpeedVac instrument and eluted in 0.1% formic acid.

The protein cross-linked complexes were analyzed using the same column and mass spectrometer as described in the section on LC-MS/MS. The peptides were separated using a segmented gradient comprising 5–25% solvent B (for 55 min) and then 25–35% solvent B (for 5 min) at a flow rate of 300 mL/min and a column temperature of 35 °C. The mass spectrometer was operated as described for LC-MS/MS, except that the target value was set to 10,000 ions.

### Data Analysis and Software Parameters (PD-XLinkX)

The raw files were analyzed using Thermo Scientific Proteome Discoverer 2.5 software, employing the XLinkX node for cross-linked peptides and the SEQUEST HT search engine for unmodified and dead-end modified peptides. The database search criteria were as follows: trypsin enzyme specificity with allowance for three missed cleavages, a precursor mass tolerance of 10 ppm, and a fragment ion mass tolerance of 0.02 Da. The search considered static alkylation of cysteine (57.021 Da), variable oxidation of methionine (15.995 Da), and variable deamidation of asparagine/glutamine (0.984 Da) as modifications.

For DMTMM cross-links, the search focused on interactions between carboxylic acids (side chains of aspartic acids and glutamic acid) and free amines (side chain of lysine) with a mass shift of −18.01056 Da. The data were compared against the target protein sequences, applying a 1% FDR criterion for protein spectral matches. The CrossLinkViewer online web tool was used for data analysis.

## Supporting Information Available

Figure S1 Purified tau and p-tau; Figure S2 Native mass spectra of tau and p-tau; Figure S3 Temperature- and concentration-dependent differences in LLPS of tau and p-tau; Figure S4 Visualization and choosing bands for mass spectrometry after cross-linking; Figure S5 RNA binding of tau and p-tau; Figure S6 Formation and stability of droplets formed of tau and p-tau in the absence or presence of poly A; Figure S7 Effect of RNase A on the formation of self-coacervation (SC) and complex coacervation (CC) droplets of p-tau; and Figure S8 Fibrillization of different concentrations of tau and p-tau in the LLPS condition (PDF)
Movie S1 Fusion process of p-tau SC droplets; Movie S2 Gravity-driven precipitation of p-tau droplets; and Movies S3–S6 TauRD accumulated in the biosensor cells subjected to p-tau_DS_, tau_DS_, p-tau_RNA_, tau_RNA_, p-tau_LLPS_, tau_LLPS_, respectively (ZIP)
