# Supplementary material for: Phosphorylation-Induced Self-Coacervation versus RNA-Assisted Complex Coacervation of Tau Proteins
Source: J Am Chem Soc. 2025 Mar 12;147(12):10172–87. doi: 10.1021/jacs.4c14728 (PMC11951079; doi:10.1021/jacs.4c14728)
Supplement: Supplementary file 1 — ja4c14728_si_001.pdf [file ja4c14728_si_001.pdf]

Phosphorylation-induced Self-coacervation versus RNA-assisted Complex Coacervation of Tau Proteins

Mohammadreza Allahyartorkaman[1,2], Ting-Hsuan Chan[2,3], Eric H.-L. Chen[2], See-Ting Ng[2], Yi-An Chen[2], Jung-Kun Wen[2], Meng-Ru Ho[2], Hsin-Yung Yen[2], Yung-Shu Kuan[3], Min-Hao Kuo[4], Rita P.-Y. Chen[2,3,5*]

[1]Taiwan International Graduate Program in Interdisciplinary Neuroscience, National Taiwan University and Academia Sinica, Taipei, 115, Taiwan

[2]Institute of Biological Chemistry, Academia Sinica, No. 128, Sec. 2, Academia Rd, Nankang, Taipei 115, Taiwan

[3]Institute of Biochemical Sciences, National Taiwan University, No. 1, Sec. 4, Roosevelt Rd, Taipei 106, Taiwan

[4]Department of Biochemistry and Molecular Biology, Michigan State University, 603 Wilson Road, Room 401, East Lansing, MI, 48824, USA

[5]Neuroscience Program of Academia Sinica, Academia Sinica, No. 128, Sec. 2, Academia Rd, Nankang, Taipei 115, Taiwan

Supplementary Materials

Figure S1. Purified tau and p-tau samples obtained by size-exclusion chromatography using a Superdex 200 10/300 GL column.

Figure S2. Native mass spectra of tau and p-tau for quantification of post-translational modifications in p-tau.

Figure S3. Temperature- and concentration-dependent differences in liquid–liquid phase separation of tau and p-tau.

Figure S4. Visualization and choosing bands for mass spectrometry after crosslinking.

Figure S5. RNA binding of tau and p-tau.

Figure S6. Formation and stability of droplets formed of tau and p-tau in the absence or presence of poly A.

Figure S7. Effect of RNase A on the formation of self-coacervation (SC) and complex coacervation (CC) droplets of p-tau.

Figure S8. Fibrillization of different concentrations of tau and p-tau in the LLPS condition.

Movie S1. Fusion process of p-tau SC droplets. The fluorescence and DIC images of a solution containing 100 μM p-tau and 1 μM Alexa Fluor 488-labeled p-tau in a solution containing 20 mM Tris and 20 mM NaCl (pH 7.4) were captured at 10-s intervals for observing droplets merging with time. The scale bar represents 20 μm.

Movie S2. Gravity-driven precipitation of p-tau droplets. p-tau (50 μM) and Alexa Fluor 488-labeled p-tau (0.5 μM) were mixed in a solution containing 20 mM Tris and 20 mM NaCl (pH 7.4). Fluorescence images were captured at different z-axis positions every 5 min.

Movie S3. TauRD accumulated in the biosensor cells subjected to dextran sulfate-treated p-tau (p-tau_{DS}). Blue: nucleus; green: tauRD aggregates. TauRD aggregates include large amorphous accumulation in the cytosol (AMO) and small speckles in the nucleus (NUC).

Movie S4. TauRD accumulated in the biosensor cells subjected to dextran sulfate-treated tau (tau_{DS}). Blue: nucleus; green: tauRD aggregates. TauRD aggregates include large amorphous accumulation in the cytosol (AMO) and small speckles in the nucleus (NUC).

Movie S5. TauRD accumulated in the biosensor cells subjected to RNA-treated p-tau (p-tau_{RNA}). Blue: nucleus; green: tauRD aggregates. Most of the tauRD aggregates are present in the nucleus (NUC), though amorphous accumulation in the cytosol (AMO) can also be found.

Movie S6. TauRD accumulated in the biosensor cells subjected to RNA-treated tau (tau_{RNA}). Blue: nucleus; green: tauRD aggregates. TauRD aggregates are present in the nucleus (NUC).

Movie S7. TauRD accumulated in the biosensor cells treated with p-tau in the LLPS condition (p-tau_{LLPS}). Blue: nucleus; green: tauRD aggregates. Most of the tauRD aggregates are present on the nuclear envelop (NE) or close to the nuclear envelop (CYT). Large amorphous accumulation in the cytosol (AMO) and small speckles in the nucleus (NUC) can also be found.

Movie S8. TauRD accumulated in the biosensor cells treated with tau in the LLPS condition (tau_{LLPS}). Blue: nucleus; green: tauRD aggregates. Most of the tauRD aggregates are present in the nucleus (NUC), though amorphous accumulation in the cytosol (AMO) can also be found.

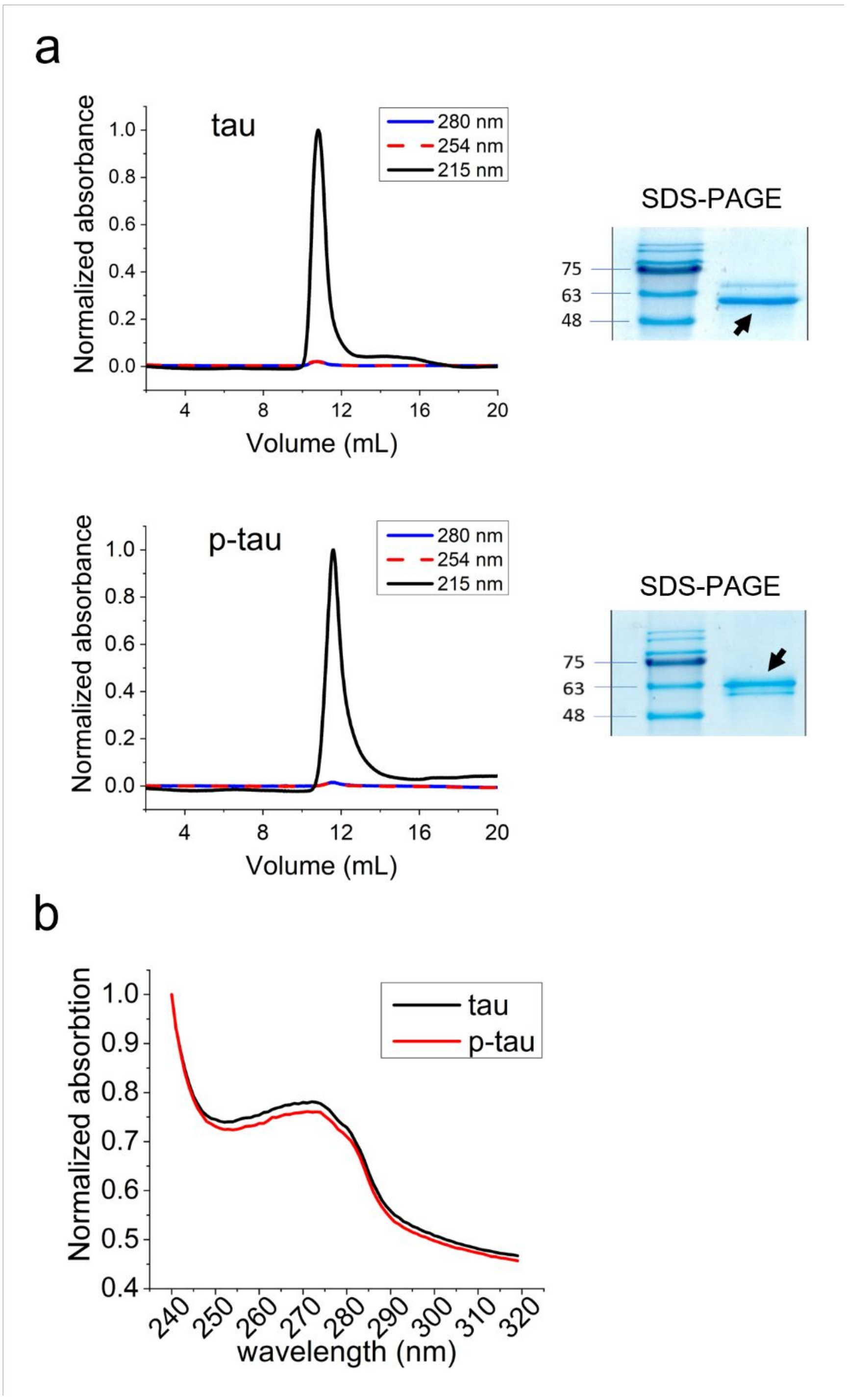

Figure S1. Purified tau and p-tau samples obtained by size-exclusion chromatography using a Superdex 200 10/300 GL column. (a) The purity of the tau and p-tau fractions was monitored by UV absorbance (left) and visualized on SDS-PAGE (right, indicated by an arrow). (b) UV spectra of tau and p-tau.

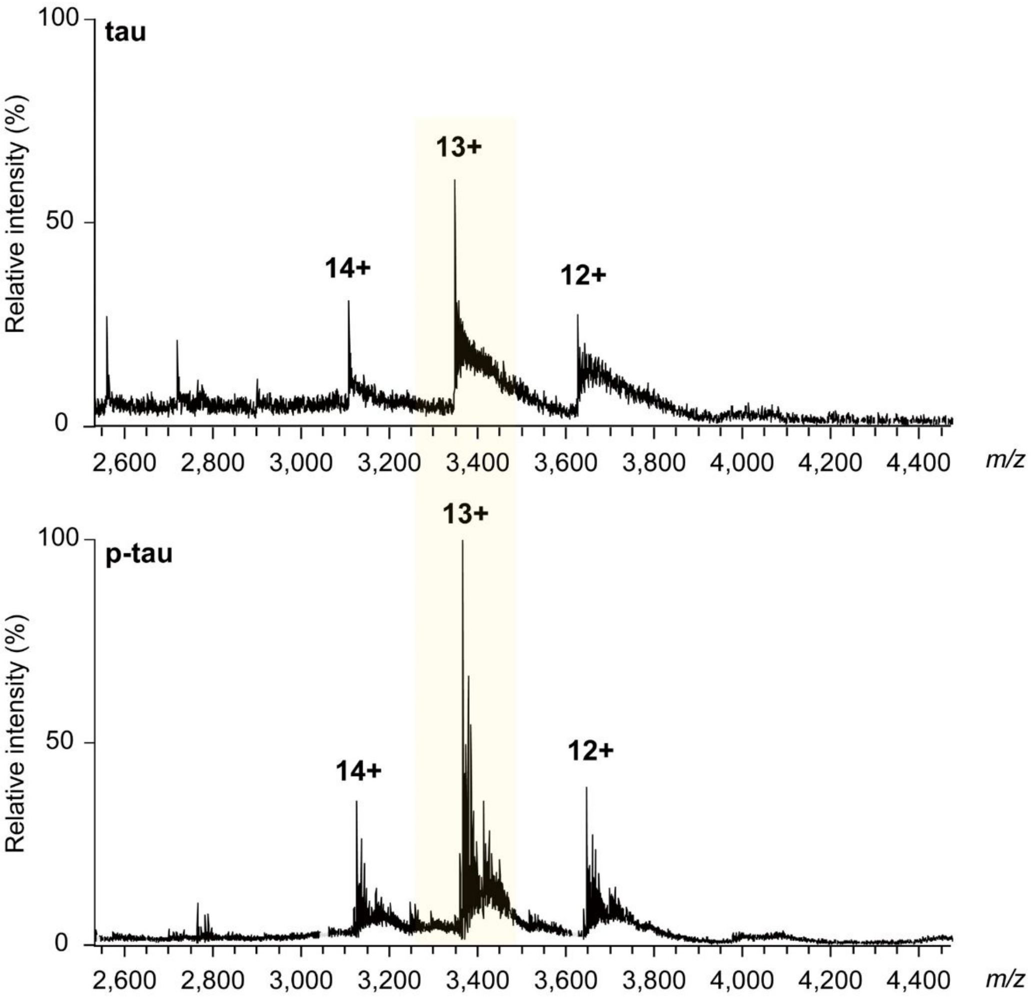

Figure S2. Native mass spectra of tau and p-tau for quantification of post-translational modifications in p-tau. The spectra of the +13 charge state are deconvoluted and shown in Figure 1d.

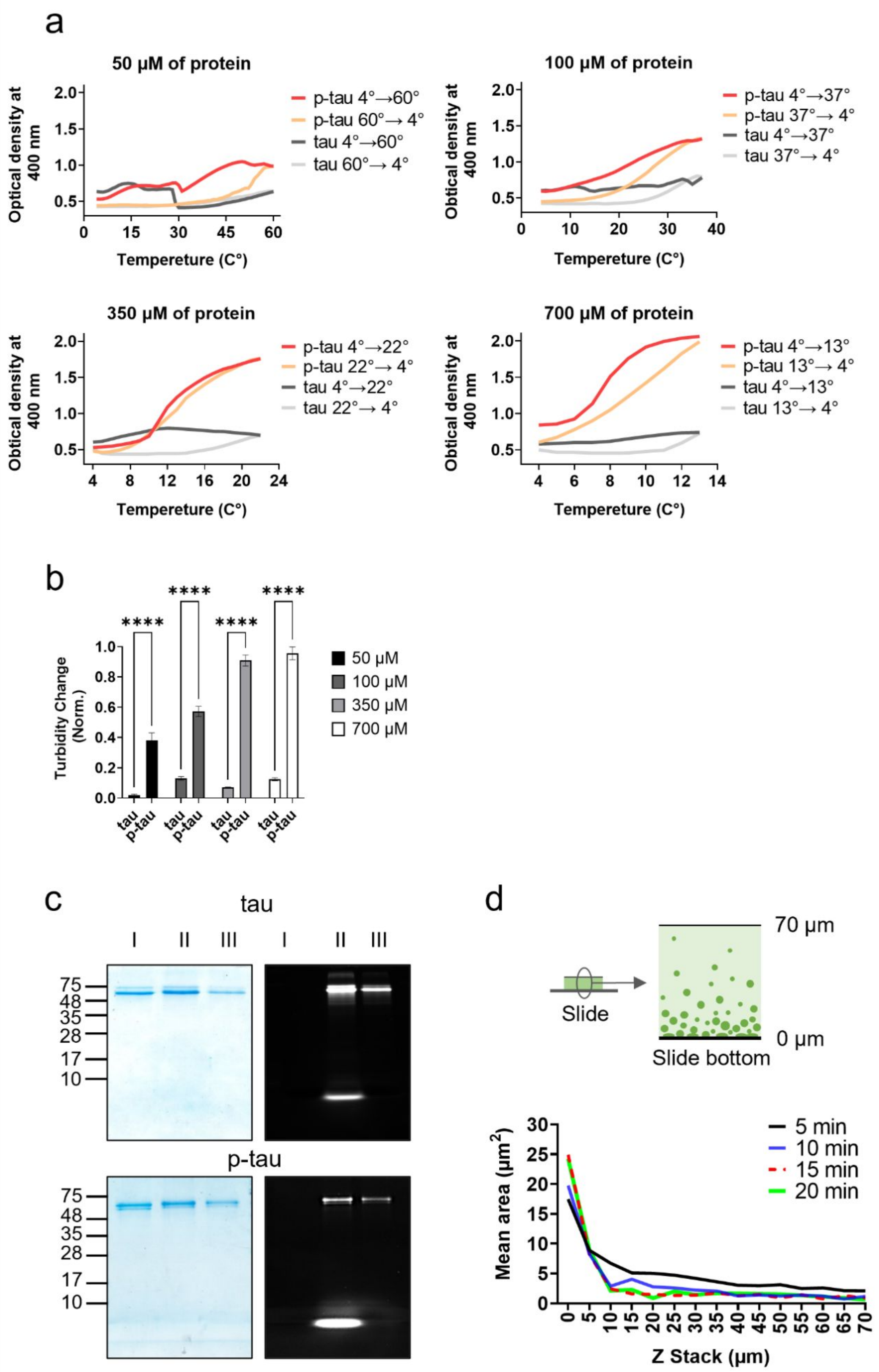
a
50 µM of protein
Optical density at 400 nm
Tempereture (C°)
p-tau 4°→60°
p-tau 60°→ 4°
tau 4°→60°
tau 60°→ 4°
100 µM of protein
p-tau 4°→37°
p-tau 37°→ 4°
tau 4°→37°
tau 37°→ 4°
350 µM of protein
Obtical density at 400 nm
p-tau 4°→22°
p-tau 22°→ 4°
tau 4°→22°
tau 22°→ 4°
700 µM of protein
p-tau 4°→13°
p-tau 13°→ 4°
tau 4°→13°
tau 13°→ 4°
b
Turbidity Change (Norm.)
****
50 µM
100 µM
350 µM
700 µM
tau
p-tau
c
tau
I II III
75 48 35 28 17 10
p-tau
d
Slide
70 µm
0 µm
Slide bottom
Mean area (µm²)
Z Stack (µm)
5 min
10 min
15 min
20 min

Figure S3. Temperature- and concentration-dependent differences in liquid–liquid phase separation of tau and p-tau. (a) The turbidity measurement at 400 nm for different concentrations of tau and p-tau with triple repeats with temperature increase and decrease without adding any crowding agents. (b) Normalized turbidity changes between the highest and lowest temperatures for different concentrations of tau and p-tau. Data are shown as mean ± SD, n = 3. A two-way ANOVA with Šídák's multiple comparisons test was used for analysis, with significance level indicated as ****$P < 0.0001$. (c) Proteins labeled with Alexa Fluor 488 were desalted using a G25 column and visualized on SDS-PAGE. The left image was stained by Coomassie Blue, and the fluorescent image (right) was illuminated using iBright FL1000 imaging systems (excitation filter 455–485 nm; emission filter 508–557 nm). The gel lanes: Lane I - protein before labeling, Lane II – labeled protein before desalting, Lane III – labeled protein after desalting. The degrees of labeling for tau and p-tau were calculated as 1.95 ± 0.03 and 1.9 ± 0.05, respectively, i.e., two dyes on one protein molecule. (d) The mean area (μm^2) of p-tau droplet precipitation at a concentration of 50 µM was determined within a time interval captured every 5 µm of the Z-stack (Movie 2).

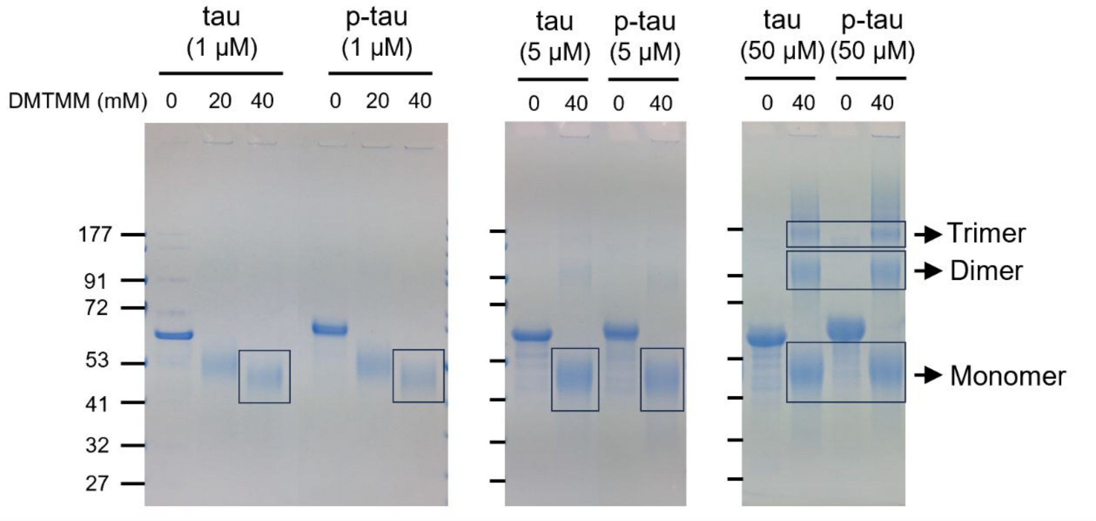

Figure S4. Visualization and choosing bands for mass spectrometry after crosslinking. Proteins were crosslinked with 4-(4,6-dimethoxy-1,3,5-triazin-2-yl)-4-methylmorpholinium chloride (DMTMM). Different concentrations (1, 5, and 50 μM) of tau and p-tau were reacted with 0 or 40 mM DMTMM for 1 h at 37°C with 700-rpm shaking. The crosslinked products were resolved on NuPAGE 4–12% Bis-Tris protein gel. Monomers, dimers, and trimers were cut and digested sequentially by Lys-C protease at 37°C for 3 h, and then with trypsin at 37°C overnight. The fragments were analyzed by mass spectrometry.

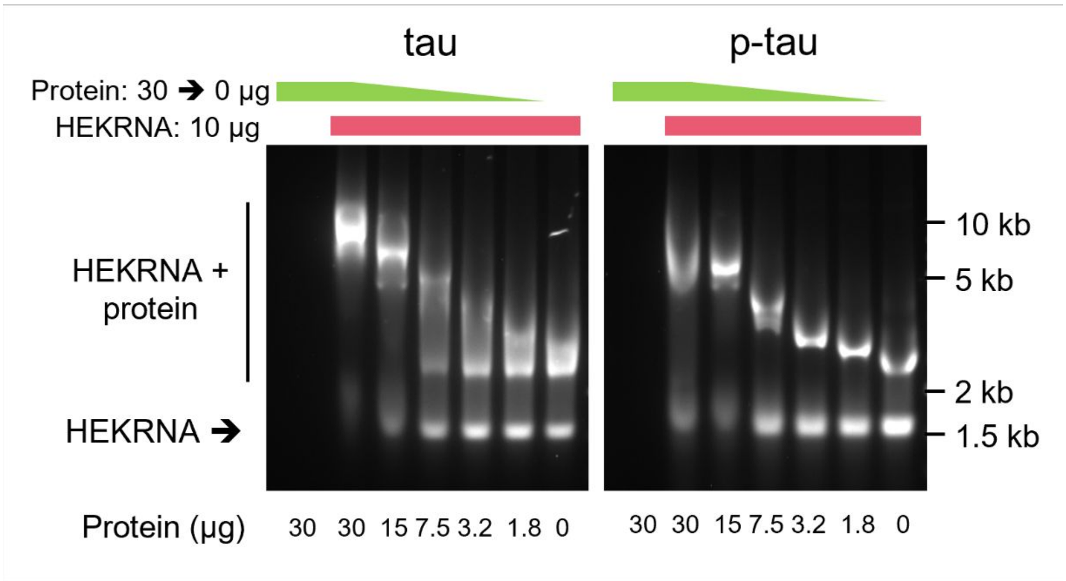

Figure S5. RNA binding of tau and p-tau. The binding of tau and p-tau to total RNA extracted from HEK293 cells (HEKRNA) was validated through electrophoretic mobility shift assay (EMSA). The EMSA confirmed that the binding ratio of RNA:protein was 1:1.5 by weight.

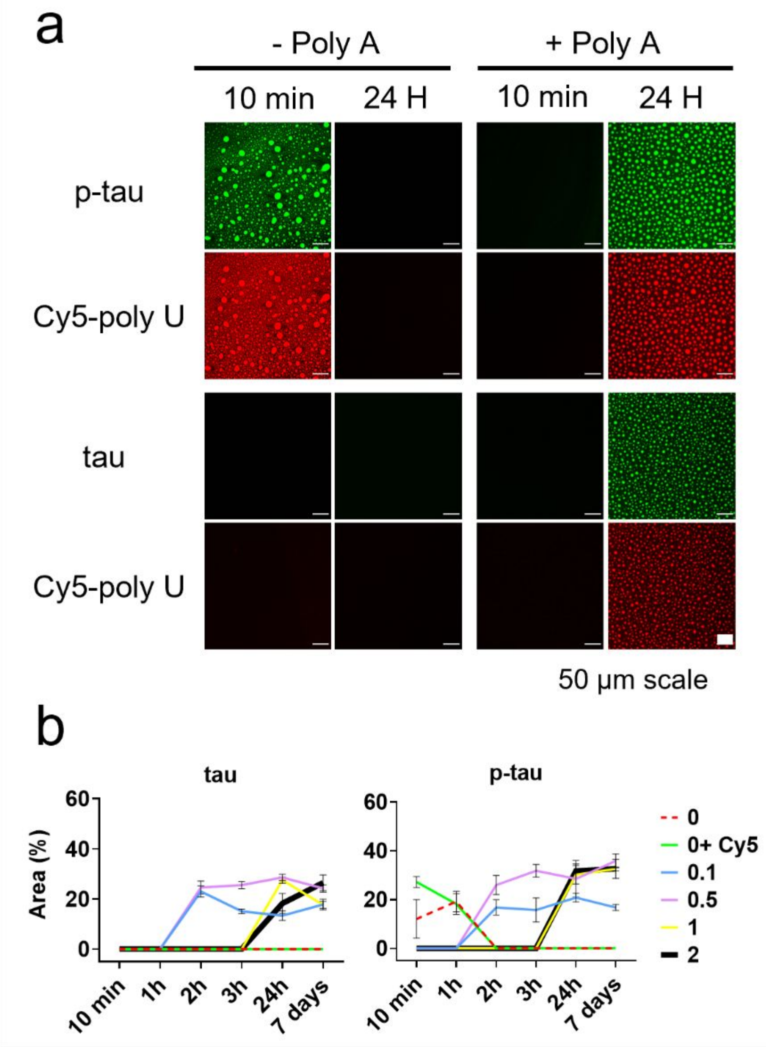

Figure S6. Formation and stability of droplets formed of tau and p-tau in the absence or presence of poly A. Poly A 40 nucleotides (nt) was mixed with tau or p-tau (50 µM) in a buffer containing 20 mM NaCl and 20 mM Tris at pH 7.4. The ratio of poly A to protein was 1:10 by weight. (a) Fluorescence imaging of tau and p-tau without (left) and with poly A (right) was acquired at 10 min and 24 h. (b) The mean area (µm²) of tau/p-tau droplets with and without poly A nt at the indicated times. Values represent mean ± SD from three independent samples. Red: no RNA; green: no poly A, but Cy5-labeled Poly U added; blue, purple, yellow, and black: poly A/protein ratio (w/w) of 0.1, 0.5, 1, and 2, respectively. To minimize the labeling effect, a 1:100 ratio of Alexa Fluor 488-labeled protein to unlabeled protein was used. The ratio of Cy5-labeled poly U (Cy5-poly U) to poly A was 1:500. Phosphorylation promoted droplet formation but could not maintain coacervation over time. In contrast, poly A inhibited the self-coacervation of p-tau. However, it induced protein condensation after 1 h, and the formed droplets sustained robustly.

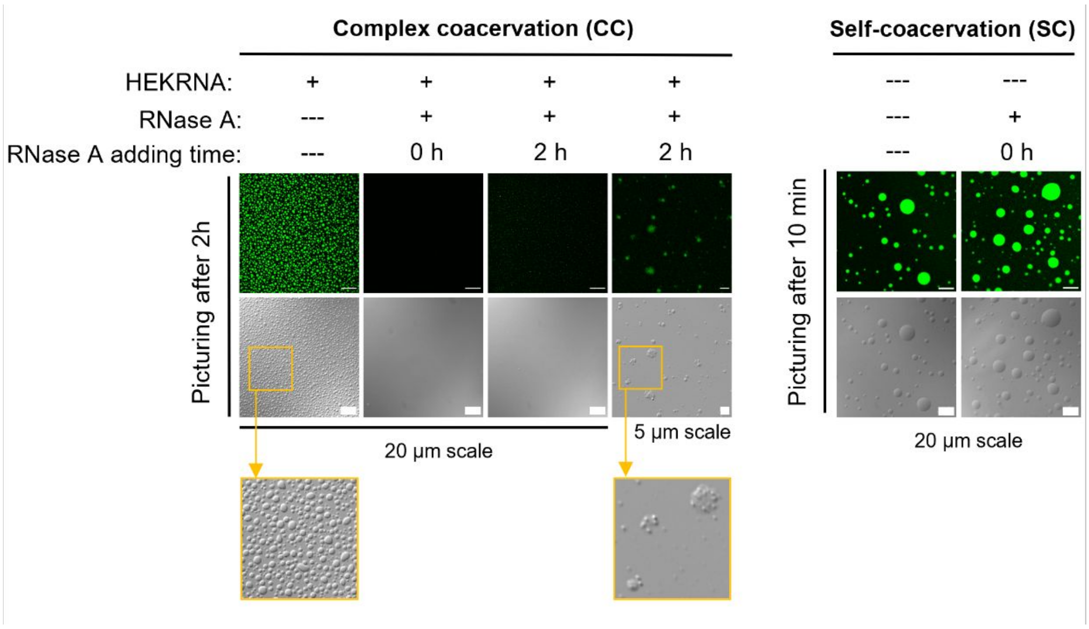

Figure S7. Effect of RNase A on the formation of self-coacervation (SC) and complex coacervation (CC) droplets of p-tau. In the solution containing 100 µg p-tau and 50 µg HEKRNA in a 20 mM Tris, 20 mM NaCl, pH 7.4 buffer, RNase A (25 µg) was added at the indicated time. RNase A completely diminished the CC of p-tau when added immediately to the p-tau/RNA mixture. On the other hand, RNA was not involved in SC; thus, no difference is observed after RNase A addition.

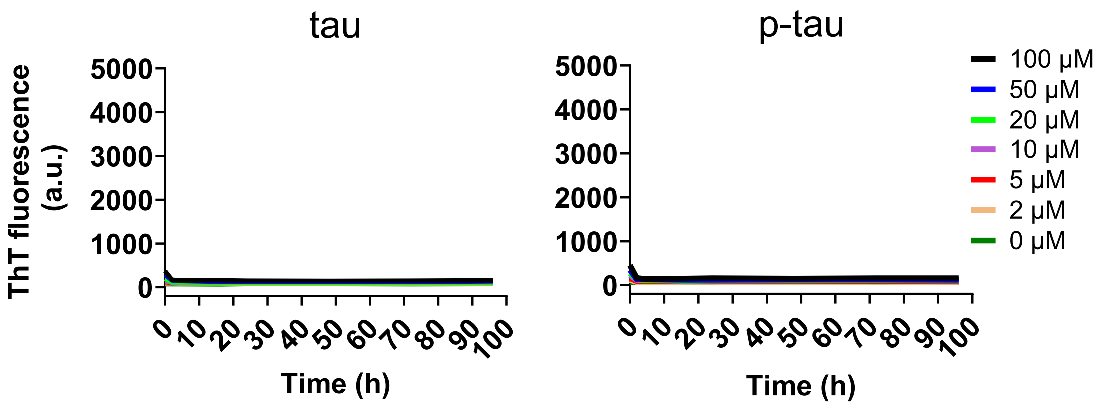

Figure S8. Fibrillization of different concentrations of tau and p-tau in the LLPS condition.
